# Control Strategy for Excipient Variability in the Quality by Design Approach Using Statistical Analysis and Predictive Model: Effect of Microcrystalline Cellulose Variability on Design Space

**DOI:** 10.3390/pharmaceutics14112416

**Published:** 2022-11-08

**Authors:** Ji Yeon Kim, Du Hyung Choi

**Affiliations:** Department of Pharmaceutical Engineering, Inje University, Gimhae-si 621-749, Gyeongnam, Korea

**Keywords:** microcrystalline cellulose, excipient variability, quality by design, design space, critical quality attributes, artificial neural networks

## Abstract

Although various quality by design (QbD) approaches have been used to establish a design space to obtain robust drug formulation and process parameters, the effect of excipient variability on the design space and drug product quality is unclear. In this study, the effect of microcrystalline cellulose (MCC) variability on drug product quality was examined using a design space for immediate-release tablets of amlodipine besylate. MCC variability was assessed by altering the manufacturer and grade. The formulation was developed by employing the QbD approach, which was optimized using a D-optimal mixture design. Using 36 different MCCs, the effect of MCC variability on the design space was assessed. The design space was shifted by different manufacturers and grades of MCC, which resulted in associations between the physicochemical properties of MCC and critical quality attributes (CQAs). The correlation between the physicochemical properties of MCCs and CQAs was assessed through a statistical analysis. A predictive model correlating the physicochemical properties of MCCs with dissolution was established using an artificial neural network (ANN). The ANN model accurately predicted dissolution with low absolute and relative errors. The present study described a comprehensive QbD approach, statistical analysis, and ANN to comprehend and manage the effect of excipient variability on the design space.

## 1. Introduction

Drug product quality is often affected by the materials used and the manufacturing process employed [1]. Manufacturing process variables, including the equipment type and settings, batch size (lab-, pilot-, and commercial-scale), operating conditions, and environmental conditions, can trigger variability and/or variation in drug product quality [2]. Mangwandi et al. showed that variations in batch size in the high-shear wet granulation process cause differences in granule mechanical properties, including granule strength, Young’s modulus, and yield stress, and these properties influence the dissolution of the drug product [3]. Suzuki et al. scaled up the blending process for mixing a lubricant by changing the blender equipment types and blending times and identified differences in the granule contact angle and hardness of drug products [4]. The quantity of active pharmaceutical ingredients (APIs) and excipients may be regarded as material variables, and the material variability caused by changes in the manufacturer and grade, lot-to-lot variation, and batch-to-batch variation might affect the physicochemical properties of the material [5]. APIs from different batches exhibit variation in physicochemical properties, including particle size distribution (PSD), agglomerates, and flowability, resulting in variability in drug product quality, such as tablet hardness and disintegration time [6]. Various studies have demonstrated that modifications in the physicochemical properties of excipients, such as PSD, moisture content, density, surface area, surface morphology, porosity, viscosity, cohesiveness, crystallinity, hygroscopicity, structural properties, and molecular properties, substantially affect drug product quality [7,8,9]. Landin et al. showed that a variation in the wood origin of microcrystalline cellulose (MCC) altered its lignin content, resulting in in vitro drug release variability [10]. Some studies demonstrated that variability in the physicochemical properties, including the viscosity of hydroxypropyl cellulose and hydroxypropyl methylcellulose, resulted in variation in the drug product quality, including in vitro drug release [11,12]. Other studies showed that variation in manufacturers of crospovidone is linked to variability in physicochemical properties, including porosity, resulting in variation in drug product quality, such as in vitro drug release [5].

Because the variation in physicochemical properties triggered by variability and/or variation in excipients can substantially affect the drug product quality, excipient variability should be thoroughly controlled to consistently yield high-quality drug products. Pharmaceutical excipients are subjected to exhaustive chemical analyses to verify their purity, and these chemical quality analyses are essential for quality control determination [13]. Although the excipient monograph specifications in various pharmacopeia provide the minimum standard of tests required, some properties are not included in pharmacopeia specifications or they may differ to a large extent within certain specifications. In particular, the function of the excipient is defined by its physicochemical properties, which are crucial for obtaining a product with optimal quality. However, physicochemical properties are not included in the excipient monograph specification. Excipient variability thus occurs and the risk of drug product quality variability increases.

To eliminate such risk of variability and/or variation when a drug product is developed or produced, the quality by design (QbD) strategy is crucial to achieving the optimal performance of robust manufacturing processes in the pharmaceutical industry as well as ensuring the safety of the final products. Based on prior knowledge and risk assessments, the variability and/or variation in material attributes are assessed to elucidate the relationship between critical material attributes (CMAs) and critical quality attributes (CQAs) in the design of experiment (DoE). This relationship is used to develop control strategies and design spaces. In previous studies employing the QbD approach, variability and/or variation in the manufacturing process and material could be well regulated [14,15,16]. Nevertheless, studies examining the impact of physicochemical property variations on drug product quality triggered by excipient variability, such as modifications in the manufacture and grade in the QbD approach, have been limited. MCC has various benefits for pharmaceutical excipients, including physiological inertness, compatibility with APIs, and security of supply. Indeed, MCC offers decent binding properties and is thus used as a diluent and binder in direct compression [17]. The effect of material properties is dependent on the amount and function of specific materials within a dosage form [18]. In general, the effect of a material decreases when it is present at a relatively low concentration within the dosage form. MCC is normally used in large quantities because it is commonly used as a binder or diluent in tablets [19]. In the present study, the effect of the variability of MCC as a diluent on the drug product quality was evaluated.

The primary objective of the present study was to assess the effect of MCC variability on the design space established by the QbD approach. The formulation of an immediate-release tablet containing amlodipine besylate was optimized using a D-optimal mixture design. The relationship between CMAs and CQAs was confirmed using empirical models in coded terms, and the design space was determined by employing multidimensional combinations and interactions. The effect of MCC variability on the design space was evaluated using optimal settings, and the design space variability was confirmed using 36 different MCCs of 13 different grades from four different manufacturers. To identify and control design space variability associated with MCC variability, a statistical analysis was also conducted. Finally, an artificial neural network (ANN) was used to develop a predictive model of dissolution.

## 2. Materials and Methods

### 2.1. Materials

Amlodipine besylate was obtained from Daewon Pharmaceutical Co., Ltd. (Seoul, Korea). MCC was purchased from the following manufacturers: DFE Pharma (Pharmacel^®^, Dusseldorf, Germany), FMC BioPolymer (Avicel^®^, Philadelphia, PA, USA), Blanver (MICROCEL^®^ MC, Farmoquímica, Sao Paulo, Brazil), and JRS Pharma GmbH & Co. KG (PROSOLV^®^SMCC (Silicified microcrystalline cellulose), VIVAPUR^®^, Heweten^®^, Rosenberg, Germany). Polyvinylpyrrolidone (PVP) was purchased from BASF AG (Kolidon^®^, Ludwigshafen, Germany). Croscarmellose sodium (CCS) was purchased from FMC BioPolymer (Ac-Di-Sol^®^, Philadelphia, PA, USA). Magnesium stearate (St-Mg) was purchased from Sigma-Aldrich Co. (St. Louis, MO, USA). All other reagents were of analytical or high-performance liquid chromatography (HPLC) grade.

### 2.2. QbD Approach for Formulation Development

#### 2.2.1. Initial Risk Assessment

The initial risk assessment was performed to clarify the risk associated with the raw material and product. Failure mode effect analysis was performed to detect CMAs and the degree of risk was quantified after obtaining the risk priority number (RPN), which was derived by multiplying the severity, probability of occurrence, and detectability scores, each of which ranged from 1 to 5. The degree of risk was determined at three levels based on the RPN as follows: low, 1–19; medium, 20–39; and high, 40–125. A high-level risk indicates that the material attribute (MA) should be controlled because it has a dramatic impact on CQAs, a medium-level risk indicates that the MA should be controlled because it has a moderate impact on CQAs, and a low-level risk indicates that the MA does not need to be controlled because it has a limited impact on CQAs [20,21].

#### 2.2.2. Experimental Design to Optimize Formulation

A D-optimal mixture design was employed, and Design-Expert^®^ software (version 13; Stat-Ease Inc., Minneapolis, MN, USA) was used to devise an experimental design. Amlodipine besylate (6.94 mg) and St-Mg (1.50 mg) were used as fixed factors. The three control factors included *x*_1_: SMCC 90 (PROSOLV^®^ SMCC 90), *x*_2_: CCS, and *x*_3_: PVP K25, and their ranges were 66.6–89.6, 1.0–15.0, and 1.0–10.0 mg, respectively. The total amount of control factors was maintained at 91.56 mg. Hardness (*y*_1_); friability (*y*_2_); dissolution at 5 (*y*_3_), 10 (*y*_4_), and 15 min (*y*_5_); assay (*y*_6_); and content uniformity (CU) (*y*_7_) were selected as response factors, and their target values are presented in Table S1. The target values of CQAs were determined using prior knowledge [22]. After the completion of 17 experiments using the D-optimal mixture design, statistical parameters, such as coefficient of determination (*R*^2^), adjusted *R*^2^, and predicted *R*^2^, were assessed to confirm the best-fit model. The adjusted *R*^2^ is used for reducing the overestimation of *R*^2^ that increases as the number of predictors increases in the model. By employing the best-fit model, the quantitative effect of CMAs on CQAs was confirmed by analysis of variance (ANOVA) and the effect was presented as a coded equation. The coefficient of the coded equation indicated the effect degree of CMAs, and a higher coefficient suggested a significant effect of CMAs on CQAs. A positive coefficient (+) signified that CMAs increase CQAs, whereas a negative coefficient (−) indicated that CMAs decrease CQAs. After employing ANOVA analysis, a design space was established with CMAs that satisfied the optimal range of CQAs.

#### 2.2.3. Tablet Preparation

The batch size was 10,000 tablets, and the weight of the powder mixture was 1000 g. Before mixing, all powders were passed through a #30 mesh sieve to remove unnecessary aggregation. The powders, excluding St-Mg, were mixed using a V-mixer (VB-3, ERWEKA GmbH, Heusenstamm, Germany) with a capacity of 3 L at 50 rpm for 10 min. After mixing, St-Mg was added to the powder bed and mixed for 5 min. The tablet was prepared by employing a single-punch tablet machine (HANDTAB-200, Ichihashi-Seiki Co., Ltd., Kyoto, Japan) using a 9 mm plane-face punch. A mass of 100 mg of powder mixture was inserted into a die and compressed at 30 MPa.

#### 2.2.4. Evaluation of CQAs

The hardness test was performed according to United States Pharmacopeia (USP) <1217> using a hardness tester (TBH 125, ERWEKA GmbH). Ten tablets were tested to obtain statistically significant results, and hardness was calculated as the mean of the measurement for 10 tablets.

The friability test was conducted according to the USP <1216> using a rotation friability tester (ERWEKA TAR 120, ERWEKA GmbH) with 30 tablets. Before the test, each tablet was weighed and tested at 25 rpm for 4 min. After the test, dust was gently removed from the tablet, which was weighed again. Friability was calculated using Equation (1):

(1) $$\mathrm{Friability}\;(\%)=\frac{w_{1\;}-{\;w}_{2}}{w_{1}}\;\times \;100,$$

where *w*_1_ is the weight before rotation and *w*_2_ is the weight after rotation.

The in vitro dissolution test was conducted with six tablets from each run order using the USP monograph method with 500 mL of dissolution medium (0.01 N hydrochloric acid) and USP Apparatus 2 guidelines (paddle method). The dissolution test was conducted using a Vision Classic 6 dissolution tester (Hanson, CA, USA) and the temperature and paddle speed were set at 37.5 °C ± 0.5 °C and 75 rpm, respectively. At predetermined times (5, 10, 15, and 30 min), 5 mL of the sample was withdrawn and filtered through a 0.45 µm nylon syringe filter (Advantec Toyo Kaisha Ltd., Tokyo, Japan). The content of the drug was analyzed using an HPLC system (Agilent, Santa Clara, CA, USA) with UV detection at 237 nm. A Symmetry C18 column (3.9 × 150 mm, 5 μm; Waters, Milford, MA, USA) was used. The mobile phase consisted of methanol, acetonitrile, and buffer (35:15:50, *v/v/v*). The buffer was prepared by adding 7.0 mL of triethylamine into a 1000 mL flask containing 900 mL of water and adjusting pH to 3.0 ± 0.1 with phosphoric acid. The flow rate and injection volume were 1.0 mL/min and 50 µL, respectively. The drug release profiles were calculated as the mean of the results for the six tablets.

Assay test was conducted according to the USP monograph method. A standard solution was prepared by diluting USP amlodipine besylate RS in a mobile phase to a concentration of 0.02 mg/mL. The sample stock solution was prepared by placing five tablets into a 500 mL volumetric flask. Then, 50 mL of the mobile phase was added to the flask and swirled to disintegrate the tablet. Following that, 300 mL of the mobile phase was added and the mixture was shaken on a reciprocating shaker for 30 min. Subsequently, the solution was diluted with the mobile phase to make up a volume of 500 mL and mixed well. The sample solution was prepared from the sample stock solution, and 0.02 mg/mL amlodipine was obtained. The sample solution was passed through a 0.45 µm nylon syringe filter. The content of amlodipine besylate was measured using HPLC (Agilent). The results were calculated using the mean of the results of five tablets. The CU test was conducted according to the USP <905> Uniformity of Dosage Units. The CU test was conducted with ten tablets and CU was calculated using Equation (2):

(2) $$\left|M-\bar{X}\right|+ks$$

where $\bar{X}$ is the sample mean as a % of label claim, *k* is the acceptability constant (2.4 in this study), *s* is the sample standard deviation, and *M* is dependent on the sample mean (note the detail of *M* value in USP).

### 2.3. Investigation of the Effect of MCC Variability on the Design Space

#### 2.3.1. Measurement of the Physicochemical Properties of MCC

Details on the physicochemical properties of MCC were obtained from the certificate of analysis (CoA) provided by the manufacturer. The CoA provided information on various physicochemical properties of MCC, such as loss on drying (LOD), pH, particle size (D10, D50, and D90), and bulk density. Details regarding physicochemical properties, such as the tapped density, true density, powder flowability, porosity, and rheological behavior of powders, were not provided in the CoA and were measured.

Tapped density was conducted according to the USP <616> Bulk Density and Tapped Density of Powder using a 100 mL mass cylinder. The powder was poured into a 100 mL mass cylinder and its weight was measured. Then, the filled cylinder was tapped 10, 500, and 1250 times, and the corresponding volumes (V10, V500, and V1250) were read. The tapped density was calculated by dividing the weight of the powder by reduced volume. Each measurement was conducted three times.

The true density of each powder was measured using a helium pycnometer (AccuPyc 1330; Micromeritics Instrument Co., Norcross, GA, USA). First, 1.57 g of the powder was weighed and poured into the sample cell. Then, the true density was calculated by filling helium gas into the sample cell and measuring the pressure in the cell. The true density of the powder was determined from the average of three measurements for each individual sample.

The flowability of powder was evaluated using the Hausner ratio (HR) and compressibility index (CI). HR and CI were calculated using Equations (3) and (4):

(3) $$HR=\frac{\rho_{\mathrm{tapped}}}{\rho_{\mathrm{bulk}}},$$

(4) $$\mathrm{CI}=\frac{\rho_{\mathrm{tapped}-}\rho_{\mathrm{bulk}}}{\rho_{\mathrm{tapped}}},$$

where *ρ_bulk_* and *ρ_tapped_* are the bulk density and tapped density, respectively.

The powder porosity was calculated using Equation (5):

(5) $$\mathrm{Powder}\;\mathrm{porosity}\;(\%)=\left[1-\frac{\rho_{\mathrm{bulk}}}{\rho_{\mathrm{true}}}\right]\text{×}100,$$

where *ρ_bulk_* and *ρ_true_* are the bulk density and true density, respectively.

To measure the dynamic flow of various MCCs, a Freeman FT4 rheometer (Freeman Technology, Malvern, UK) was used. The test was conducted using a 25 mm × 25 mL split vessel and a 23.5 × 6 mm blade. Before the test, conditioning cycles were repeatedly performed using the instrument’s conditioning methodology [23]. Before initial conditioning, approximately 11 g of each powder was loaded into the cell. During conditioning cycles, the blade was moved downward and upward at a blade tip speed and helix angle of 40 mm/s and −5°, respectively [24]. After the initial conditioning, any material above the bed height of the cell was removed. The test cycle was conducted after conditioning, and 11 tests were performed. Initially, seven runs were conducted with a blade tip speed of 100 mm/s, and three additional tests were conducted at speeds of 70, 40, and 10 mm/s [25]. Using an FT4 rheometer, basic flowability energy (BFE), the stability index (SI), the flow rate index (FRI), specific energy (SE), and conditioned bulk density (CBD) were obtained. BFE, SI, FRI, SE, and CBD were calculated using Equations (6)–(10) [26]:

(6) BFE (mJ)=Energy test 7,

(7) $$\mathrm{SI}=\frac{\mathrm{Energy}\;\mathrm{test}\;7}{\mathrm{Energy}\;\mathrm{test}\;1},$$

(8) $$\mathrm{FRI}=\frac{\mathrm{Energy}\;\mathrm{test}\;11}{\mathrm{Energy}\;\mathrm{test}\;8},$$

(9) $$\mathrm{SE}\;(\mathrm{mJ}/g)=\frac{\mathrm{Up}\;\mathrm{Energy}\;\mathrm{cycle}\;6+\mathrm{Up}\;\mathrm{Energy}\;\mathrm{cycle}\;7}{2\text{×}\mathrm{Split}\mathrm{mass}},$$

(10) $$\mathrm{CBD}\;(g/\mathrm{mL})=\frac{\mathrm{Split}\;\mathrm{mass}}{\mathrm{Split}\;\mathrm{volume}},$$

#### 2.3.2. Statistical Analysis of the Physicochemical Properties of MCC and CQAs

MCC variability and the correlations of the physicochemical properties of MCC with CQAs were investigated using principal component analysis (PCA) and Pearson correlation coefficient (PCC), respectively. PCA and PCC were performed using SIMCA^©^ software (Sartorius Stedim Biotech., version 15, Umeå, Sweden) and Origin 2022 software (OriginLab, Northampton, MA, USA), respectively. PCA is a multivariate analysis method that transforms numerous datasets into a new system of variables known as principal components (PCs), thereby facilitating data interpretation [27]. The axis with the highest variance is identified as the first PC, and the axis with the second largest variance is identified as the second PC [28]. PCC quantifies the linear relationship between two variables (X and Y) and ranges from −1 to +1 [27]. In the present study, X variables included the physicochemical properties of MCC, whereas the Y variables included CQAs. PCC of −1 indicates a negative linear relationship, i.e., Y decreases as X increases. PCC of +1 indicates a positive linear relationship, i.e., Y increases as X increases. PCC of 0 indicates no correlation between two variables. PCC was calculated using Equation (11):

(11) $$r=\frac{\sum_{i}^{n}\left(X_{i}-\bar{X}\right)\left(Y_{i}-\bar{Y}\right)}{\sqrt{\sum_{i}^{n}{\left(X_{i}-\bar{X}\right)}^{2}}\sqrt{\sum_{i}^{n}{\left(Y_{i}-\bar{Y}\right)}^{2}}}$$

where *r* is the strength of the linear relationship between *X* and *Y*, n is the number of instances, $\bar{X}$ is the average of *X* samples, and $\bar{Y}$ is the average of *Y* samples. 

#### 2.3.3. ANN Modeling

ANN modeling to predict dissolution was performed using a feed-forward neural network, which was modeled using the Neural Network Toolbox in MATLAB (R2018a, Mathworks, Inc., Natick, MA, USA). A non-linear autoregressive with external input network involving a hidden layer with 10 neurons and an output layer with three neurons was created in MATLAB. The prediction ability and fit of the model were confirmed by calculating the mean square error (MSE) and *R*^2^. MSE and *R*^2^ were calculated using Equations (12) and (13) [29,30]:

(12) $$\mathrm{MSE}=\frac{\sum_{i=1}^{N}{{(Y}_{i}^{\mathrm{pred}}-Y_{i}^{\exp})}^{2}}{N},$$

(13) $$R^{2}=\frac{\sum_{i=1}^{N}{\left(Y_{i}^{exp}-Y_{i}^{pred}\right)}^{2}}{\sum_{i=1}^{N}{\left(Y_{i}^{exp}-{\bar{Y}}_{ave}^{exp}\right)}^{2}},$$

where *N* is the number of training data, ${\bar{Y}}_{i}^{pred}$ is the predicted output, ${\bar{Y}}_{i}^{exp}$ is the experimental results, ${\bar{Y}}_{ave}^{pred}$ is the average predicted output, and ${\bar{Y}}_{ave}^{exp}$ is the average experimental results.

## 3. Results and Discussion

### 3.1. QbD Approach for Optimizing the Formulation

#### 3.1.1. Initial Risk Assessment for Formulation Development

The quality target product profile (QTPP) involved the elements associated with quality, safety, and efficacy, such as the route of administration, dosage form, and dosage strength. Considering the reference drug properties, label, and targeted patient population, the target values were established. Table S2 presents the QTPP and CQAs for amlodipine tablets. Since assays and CU can affect safety and efficacy, and the therapeutic efficacy depends on the accuracy of the API content, an assay and CU were chosen as CQAs. Since the drug dissolution rate may be directly associated with bioavailability and drug delivery, dissolution was thus selected as a CQA. Because inadequate tablet hardness may affect safety and efficacy, it should be adequately robust so as to avoid breaking during routine handling, and it should not have a considerable impact on dissolution. Hardness was thus selected as a CQA. Friability is an additional routine test based on the compendial requirements for tablets, and drug loss attributable to abrasion of the drug affects safety and efficacy, indicating that it should be minimized. Friability should therefore be evaluated throughout formulation development. An assay, CU, dissolution, hardness, and friability were selected as CQAs, and they were assessed as response factors for formulation development.

The initial risk assessment was conducted to identify CMAs, and the result of the initial risk assessment is presented in Table S3. Severity assesses the implications of the effect of MAs on CQAs and how this effect of MAs may affect CQAs. The probability of occurrence, a possibility of failure, is the probability that MAs cannot meet the target criteria of CQAs. Detectability is the capability to detect failure caused by MAs on CQAs [31]. The detectability score was set according to the detection/control method: HPLC, 4; dissolution tester, 4; hardness tester, 3; and friability tester, 3.

The risks of SMCC 90 were classified as a medium risk for the assay (RPN: 36), medium risk for CU (RPN: 36), high risk for dissolution (RPN: 48), high risk for hardness (RPN: 48), and high risk for friability (RPN: 48). The cause of risk was linked to both the severity and probability of occurrence scores. The effect of the raw material on CQAs depends on its content in the drug product, and the content of SMCC 90 used as a diluent comprises a large part of the tablet content. In addition, because MCC has a porous structure, water effectively penetrates into the hydrophilic tablet matrix to induce swelling and the disintegration of the tablet, thereby substantially affecting dissolution [32]. Moreover, MCC is an excipient with great compressibility, having a substantial impact on hardness and friability [33]. The probability of failure of SMCC 90 for assay and CU may occur rarely. The failure probabilities of SMCC 90 for dissolution, hardness, and friability are high due to operator error, equipment failure, and material variation in SMCC 90; therefore, appropriate management of these parameters is needed during the process to avoid failure.

The risks of CCS were classified as a low risk for the assay (RPN: 8), low risk for CU (RPN: 8), high risk for dissolution (RPN: 100), low risk for hardness (RPN: 18), and low risk for friability (RPN: 18). Since the proportion of CCS is low in a tablet, its influence on the assay, CU, hardness, and friability is also low. However, as a disintegrant, CCS has water-absorbing and swelling properties [34,35], thereby significantly affecting drug release. The probability of failure of CCS for the assay and CU may occur but is unlikely to happen. The probability of failure of CCS for dissolution is high due to operator error, equipment failure, and material variation in CCS, requiring proper management. The potential failure of CCS with respect to hardness and friability may occur rarely.

The risks of PVP K25 were classified as a low risk for the assay (RPN: 8), low risk for CU (RPN: 8), high risk for dissolution (RPN: 100), high risk for hardness (RPN: 48), and high risk for friability (RPN: 48). The content of PVP K25 is low in a tablet, so its influence on the assay and CU is also low. However, PVP K25 used as a binder significantly affects tablet dissolution, hardness, and friability because PVP is directly associated with binding force. Moreover, when PVP K25 comes into contact with water, its viscosity increases, resulting in increased bonding strength with other ingredients in the tablet, whereas the hydration rate is reduced, resulting in changes in dissolution [34]. The failure of PVP K25 for the assay and CU may occur infrequently. The probability of failure of PVP K25 for dissolution, hardness, and friability is high due to operator error, equipment failure, and material variation in PVP K25, requiring proper management to achieve the target criteria of CQAs.

The risks of St-Mg were classified as low risk for assay (RPN: 8), low risk for CU (RPN: 16), low risk for dissolution (RPN: 8), low risk for hardness (RPN: 6), and low risk for friability (RPN: 6). Although long lubrication times can affect the tablet quality due to particle delamination, the content of St-Mg in the tablet content is low and therefore has less effect on CQAs. The potential failure of CQAs due to St-Mg is low and is well managed at the lab scale.

#### 3.1.2. Effect of CMAs on CQAs

Table 1 presents the results of DoE and ANOVA. As presented in Table 1, the results of the assay and CU fulfilled the targeted values of them and were thus excluded from the statistical analysis. Based on the data in Table 1, the *p*-values of all models were less than 0.05, suggesting that the model was significant for all responses. The effects of CMAs on CQAs (hardness; friability; and dissolution at 5, 10, and 15 min) are expressed as coded equations with mathematical models. As shown in Table 1, *R*^2^, adjusted *R*^2^, and predicted *R*^2^ of all response factors were high, indicating that all the models were fit. In addition, the difference between adjusted *R*^2^ and predicted *R*^2^ was less than 0.2, which indicates these two statistical parameters were considered to be in reasonable agreement. As presented in Table 1, the mutual interaction between SMCC 90 (*x*_1_) and PVP K25 (*x*_3_) had a positive effect on hardness, and a negative effect on friability. Dissolution was affected by the mutual interactions between SMCC 90 (*x*_1_) and CCS (*x*_2_) and between CCS (*x*_2_) and PVP K25 (*x_3_*). In particular, the mutual interaction between CCS (*x*_2_) and PVP K25 (*x*_3_) had a significantly positive impact on dissolution. Nevertheless, the main effect of PVP K25 on dissolution was negative. Plastic deformation is triggered by a particle bed within the cavity inside the die during the compression process, and greater plastic deformation produces a mechanically stronger tablet. Because MCC primarily exhibits plastic deformation during compression [36], when MCC was compressed, its binding area increased, leading to an increase in tablet hardness without increasing tablet friability [34]. In general, PVP is utilized as a binder in the wet granulation and direct compression process because of its high binding strength. In the direct compression process, the moisture content of the ingredients is essential, and in the case of PVP, the water contained in PVP can increase hardness because it provides the bonding force between the particles [37]. In addition, PVP ensures the desired tablet hardness without increasing the tablet friability, and consequently, SMCC 90 and PVP K25 can enhance hardness and reduce friability. When PVP K25 interacts with water, its viscosity increases, and the bonding strength of other ingredients in the tablet also increases [37]. In contrast, the rate of hydration is decreased, and drug release might be delayed [34]. PVP K25 thus exerted a detrimental effect on dissolution. CCS triggers tablet disintegration by generating pores in the tablet matrix via the relaxation of cellulose fibers and by inducing water penetration and the breakdown of hydrogen bonds [35]. PVP K25 and CCS can thus decrease and increase drug release, respectively.

#### 3.1.3. Establishment of the Optimal Setting and Robust Design Space

**Figure 1:**
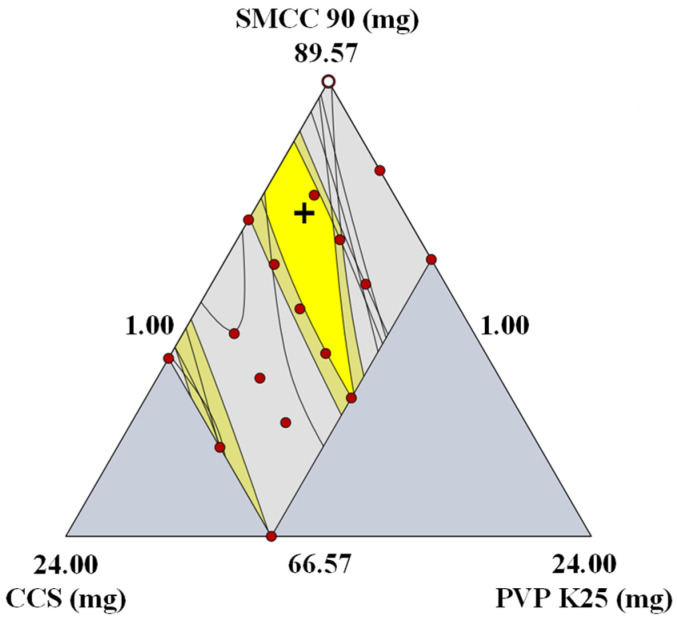
Design space in formulation development. The yellow area corresponds to the 95% confidence interval that satisfies the target values of CQAs. (SMCC, silicified microcrystalline cellulose; CCS, croscarmellose sodium; PVP, polyvinylpyrrolidone).

Based on the result of the DoE, a design space was established by combining the response factors to produce an optimal region. A design space graphically represents the relationship between the control factors and response factors and highlights the ranges of the control factors within which CQAs maintain consistent quality [38]. The optimal ranges of CQAs are presented in Table S1. The results for the assay and CU were excluded in the design space analysis because they satisfied the target values. Figure 1 presents the design space optimizing for the optimal formulation. The dark yellow region corresponds to the region satisfying the criteria, although there is a part of an internal estimate that does not satisfy these criteria. The gray region indicates the region that did not fit the optimization criteria, the red points indicate the experimental points, and the crosshair presents the optimal setting.

Based on these data, the acceptable CMA ranges for achieving and maintaining drug product quality within the limits defined in QTPP were as follows: SMCC 90, 74.2–86.4 mg; CCS, 4.5–7.6 mg; and PVP K25, 1.2–6.5 mg. To verify the robustness of the design space, a validation process was performed. The tablet for the validation test was prepared using the optimal conditions. Table S4 presents the optimal settings, target values, absolute biases, and relative biases. Absolute biases represent the difference between the estimated and target values, and relative biases represent the absolute biases divided by the estimated values. The absolute biases of all response factors were 0.01–6.14, and the relative biases of all response factors were 1.86–7.69%. Dissolution at 15 min had high absolute (6.14) and relative biases (6.81%), but the tablet for the validation test satisfied the optimal range of dissolution at 15 min. In addition, the absolute bias (0.01) of friability was low, but the relative bias (7.69%) was high. However, the optimal range of friability was satisfied. All response factors satisfied the optimal range and showed acceptable absolute and relative biases; hence, it is expected to produce drug products of the desired quality in the design space.

### 3.2. Effect of MCC Variability on Drug Product Quality and the Design Space

#### 3.2.1. Risk Assessment for MCC Physicochemical Properties

As globalization and international trade expand, it should develop harmonized compendial requirements to ensure consistent drug product quality [39]. The harmonization of pharmaceutical requirements reduces the manufacturer’s burden of performing different test methods and using different criteria to satisfy the pharmacopeial requirements of different regions. The Pharmacopeial Discussion Group (PDG) makes an effort to harmonize the pharmacopeial standards in three regions (USP–National Formulary (NF), European Pharmacopoeia (Ph. Eur.), and Japanese Pharmacopeia (JP)), and several harmonized general chapters and an excipient monograph were published. The harmonization of pharmacopeial standards by the PDG can reduce the burden on manufacturers and strengthen individual pharmacopeias by establishing robust monographs. However, the harmonization of requirements and standards of worldwide pharmacopeias is still lacking. Since the PDG harmonization processes consider only the three pharmacopeias (USP–NF, Ph. Eur., and JP), several critical national pharmacopeias are not included in the harmonization process, resulting in differences in monograph requirements for excipients, drug substances, and drug products [40].

In addition to compendial requirements’ harmonization, it needs to focus on the identification of excipient attributes that could potentially affect CQAs. Identifying excipient attributes in drug development which apply the QbD approach is crucial because QbD emphasizes understanding the role and effect of excipient attributes on CQAs. The object of drug product development by applying the QbD approach is to design high-quality drug products by understanding and controlling CMAs that could affect CQAs and to develop a drug product manufacturing process that consistently produces the intended product quality. The pharmaceutical excipients’ manufacturing and supplier comply with the compendial standards. However, the effects of excipient attributes on CQAs depend on the formulation and process of drug products, and excipient attributes not evaluated in compendial standards may also affect CQAs. Generally, pharmacopeia standards focus on identification, quality, purity, packaging, and labeling [41]; however, it is not enough to satisfy pharmacopeia specifications because the functionality of an excipient is determined by its physicochemical properties [13]. It is thus essential to comprehensively assess the physicochemical properties of the excipient, which can affect the drug product quality. A risk assessment to identify the physicochemical properties of MCC as a potential risk for CQAs was conducted following the same method described in Section 2.2.1. Table S5 presents the MCC monograph specification according to different pharmacopeia and manufacturers. In the present study, the following four pharmacopeias were compared: USP–NF, Ph. Eur., JP, and Korean Pharmacopeia.

Based on MCC monograph specifications in the pharmacopeia and from the manufacturers, an initial risk assessment was performed. Table S6 presents the risk assessment for MCC. Although true density is not contained in pharmacopeia monograph and manufacturer specifications, it was evaluated in the risk assessment because it may affect the drug quality [42]. The following can affect the stability, efficacy, and performance of drug products: appearance, identification A and B, residue on ignition, residual solvent, solubility in an ammoniacal solution of copper tetramine R, conductivity, water-soluble substances, ether-soluble substances, heavy metals, mercury, cadmium, lead, arsenic, starch, total aerobic microbial count, total combined mold and yeasts count, *Staphylococcus aureus*, *Pseudomonas aeruginosa*, *Escherichia coli*, *Salmonella* species, *Enterobacteriaceae*, *Coliform* species, and packaging and storage [43,44]. However, they were classified as low risk factors because they displayed a limited potential risk for affecting the CQAs considered in the formulation development, including the assay, CU, dissolution, hardness, and friability [7]. Because the degree of polymerization of MCC could have a major impact on tablet tensile strength and compressibility [45], the degree of polymerization might considerably affect the assay, CU, dissolution, hardness, and friability. Because solubility is associated with dissolution, it was considered a high risk factor for dissolution. The solubility and dissolution of amlodipine besylate can be affected by pH [46,47], and pH was thus classified as a medium risk factor for dissolution. In general, moisture in the powder can influence several powder properties, including cohesion and flowability [48,49]. Because flowability might influence the assay and CU, LOD was believed to have an impact on the assay and CU. In addition, as moisture content can affect the physicochemical properties of the tablet, LOD was considered to have a significant impact on dissolution, hardness, and friability [49,50]. Moreover, bulk density, tapped density, and PSD were considered high risk factors, whereas powder flow was considered a medium risk factor for the assay, CU, dissolution, hardness, and friability. These properties are associated with each other and with flowability. Particle size and PSD considerably impact flowability, CU, compressibility, and dissolution, which can affect a drug product’s safety and efficacy [51]. Flowability is an essential property, and inadequate flowability adversely affects the properties of a tablet, including hardness, friability, and dissolution, because the powder mixture will not effectively fill the die in the tableting process [52,53]. The true density of MCC is associated with its water content [54], and this is associated with tablet porosity. Because this physicochemical property can affect tablet properties, including tensile strength and compatibility [55], true density can affect tablet hardness and friability.

#### 3.2.2. Effect of MCC Variability on Drug Product Quality

**Figure 2:**
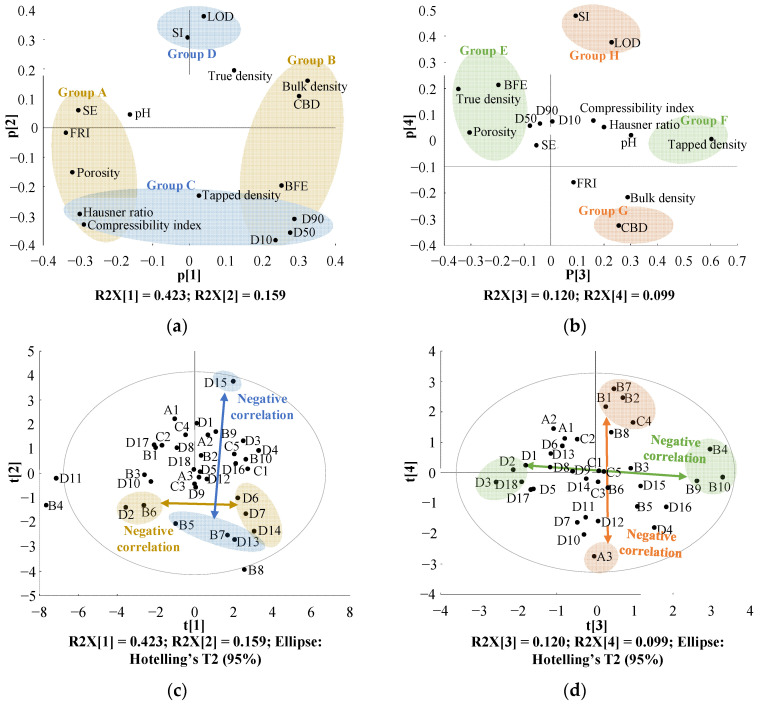
Result of principal component analysis; (**a**) Loading plot with PC1 and PC2; (**b**) Loading plot with PC3 and PC4; (**c**) Score plot with PC1 and PC2; (**d**) Score plot with PC3 and PC4. The yellow area indicates physicochemical properties significantly affecting PC1, the blue area denotes the physicochemical properties significantly affecting PC2, the light green area indicates the physicochemical properties significantly affecting PC3, and the orange area indicates the physicochemical properties significantly affecting PC4. (PC, principal component; LOD, loss on drying; BFE, basic flowability energy; SI, stability index; FRI, flow rate index; SE, specific energy; CBD, conditioned bulk density.)

To assess the variations among manufacturers and grades with respect to the physicochemical properties of MCC, a PCA model was established. The model was fitted with four PCs, which accounted for 80.1% of the variability in the assessed physicochemical properties of MCC. Specifically, the first, second, third, and fourth PCs accounted for 42.3, 15.9, 12.0, and 9.9%, respectively, of the variability. Figure 2a demonstrates the loading plot with PC1 and PC2, and Figure 2b presents the loading plot with PC3 and PC4. Figure 2a highlights the fact that the variability of PC1 was predominantly dominated by powder flowabilities, including SE, FRI, porosity, HR, CI, BFE, PSD (D10, D50, and D90), bulk density, and CBD. As presented in Figure 2a, a negative correlation between group A (SE, FRI, porosity, HR, and CI) and group B (BFE, PSD, bulk density, and CBD) was noted in PC1. PC2 was predominantly affected by LOD, SI, HR, CI, tapped density, and PSD (D10, D50, and D90). Furthermore, a negative correlation between group C (HR, CI, tapped density, and PSD) and group D (LOD and SI) was noted in PC2. Figure 2b reveals the fact that the variability in PC3 was dominated by true density, BFE, porosity, and tapped density. Moreover, there was a negative correlation between group E (true density, BFE, and porosity) and group F (tapped density) in PC3. PC4 was predominantly affected by SI, LOD, and CBD. As presented in Figure 2b, a negative correlation existed between group G (CBD) and group H (SI and LOD) in PC4.

The score plots (Figure 2c,d) correspond to the MCC variability among manufacturers and grades. Each MCC has an abbreviation based on the manufacturer as follows: A (DFE Pharma), B (FMC BioPolymer), C (Blanver), and D (JRS Pharma GmbH & Co. KG); the full abbreviation and the physicochemical properties of each MCC are presented in Table 2. As indicated in Figure 2c, in PC1, the variability of physicochemical properties was predominantly associated with the modification of grades. D2 and B6 exhibited negative loading values in PC1, whereas D6, D7, and D14 displayed positive loading values in PC1. When MCC presents a positive score in PC1, it is expected to present high BFE, PSD, bulk density, and CBD. By contrast, when MCC presents a negative score in PC1, it is expected to present low BFE, PSD, bulk density, and CBD. As presented in Figure 2c, variability in the physicochemical properties was noted because of changes in the MCC grade in PC2. In PC2, D15 exhibited a positive loading value in PC2, whereas B5, B7, and D13 had negative loading scores in PC2. As demonstrated in Figure 2a, MCC with a positive score in PC2 is expected to present high LOD and SI and low HR, CI, tapped density, and PSD. Conversely, MCC with a negative score in PC2 is expected to present low LOD and SI and high HR, CI, tapped density, and PSD.

As illustrated in Figure 2d, in PC3, variability in the physicochemical properties was associated with a change in the MCC grade. D2, D3, and D18 exhibited negative scores in PC3, whereas B4, B9, and B10 showed positive scores. MCC with a positive score in PC3 is expected to present high tapped density and low BFE, true density, and porosity. In contrast, MCC with a negative score in PC3 exhibited low tapped density and high BFE, true density, and porosity. As presented in Figure 2d, the variability in physicochemical properties was noted with shifts in the MCC grade and manufacturer in PC4. B1, B2, B7, and C4 presented positive loading scores, whereas A3 had a negative loading score in PC4. As demonstrated in Figure 2b, MCC with a positive score in PC4 is expected to present high LOD and SI and low CBD. Contrarily, MCC with a negative score in PC4 exhibited low LOD and SI and high CBD.

**Figure 3:**
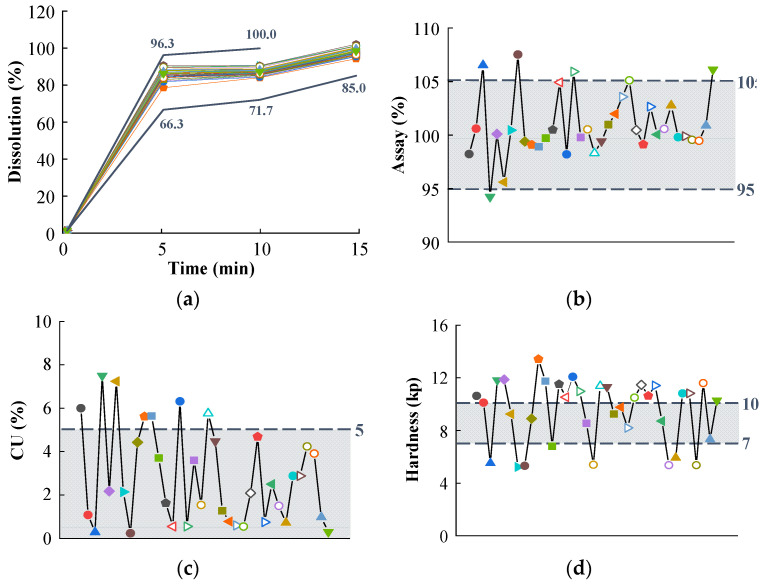

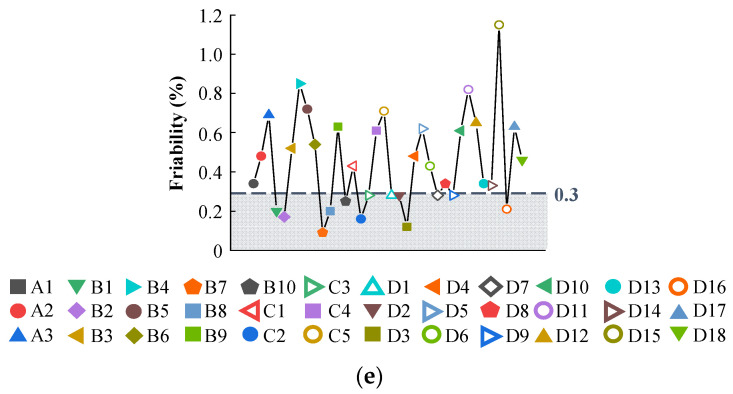
The CQAs of 36 different MCCs; (**a**) Dissolution; (**b**) Assay; (**c**) Content uniformity; (**d**) Hardness; (**e**) Friability. The gray dotted lines and box indicate the optimal ranges of CQAs. (CQA, critical quality attribute; MCC, microcrystalline cellulose).

To validate the impact of MCC variability on the design space, CQAs were determined by modifying the MCC manufacturer and grade in the optimal settings identified in the QbD approach. Using 36 MCC samples (Table 2), the tablet was prepared using the optimal formulation and compressed as described in Section 2.2.3. Figure 3 presents the results of the CQA measurements based on the MCC variability, and significant differences in CQAs were identified. As shown in Figure 3, the optimal dissolution ranges were fulfilled, but the optimal ranges for the assay, CU, hardness, and friability were not satisfied. In particular, MCC did not meet the optimal ranges of hardness and friability caused by the variation in physicochemical properties associated with differences in manufacturers and grades. The hardness and friability of D3 (MCC used in formulation development) were 9.25 kp and 0.12%, respectively. B4 displayed the lowest hardness (5.23 kp), whereas D15 displayed the highest friability (1.15%). The largest variations in the physicochemical properties of MCC were noted between D3 and B4 and between D3 and D15. D3 is a combination of MCC and colloidal silicon dioxide. D3 exhibits higher bulk density, better flow properties, and improved CI as compared with those of the common MCC types [56]. Owing to its properties, D3 is predominantly utilized for the direct compression process. B4 exhibits increased compactibility, unacceptable tablet weight variability, and extremely low disintegrating properties compared with those of the other grades [57]. Owing to its properties, B4 is predominantly utilized in the roller compaction process. As presented in Table 2, the CIs of D3 and B4 were 18.60% and 60.00%, respectively. B4 thus had poor flowability, whereas D3 exhibited decent flowability. Owing to its low flowability, B4 displayed less hardness than D3. D15 is a 301 grade of MCC, featuring the same quality as the 101 grades, but it increased bulk density and improved flowability. The bulk densities of D3 and D15 were 0.35 g/mL and 0.40 g/mL, respectively. The higher bulk density could decrease tensile strength and increase friability [58]. As D15 presented higher bulk density than D3, it exhibited higher friability.

#### 3.2.3. Effect of A Changes in the Manufacturer on Design Space

**Figure 4:**
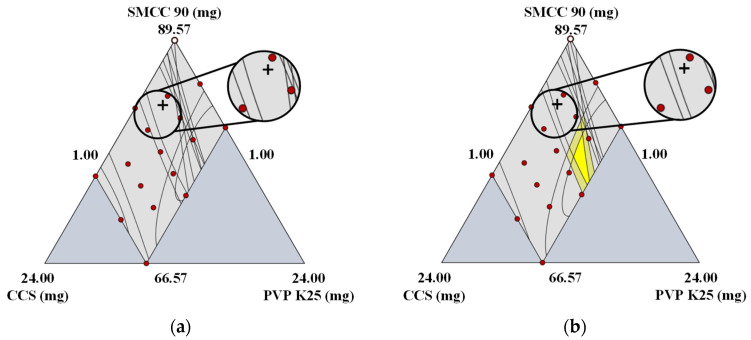
Design space variability resulting from changes in the manufacturer; (**a**) Design space using A3; (**b**) Design space using C4. The crosshair indicates the optimal setting of formulation development. (SMCC, silicified microcrystalline cellulose; CCS, croscarmellose sodium; PVP, polyvinylpyrrolidone).

**Figure 5:**
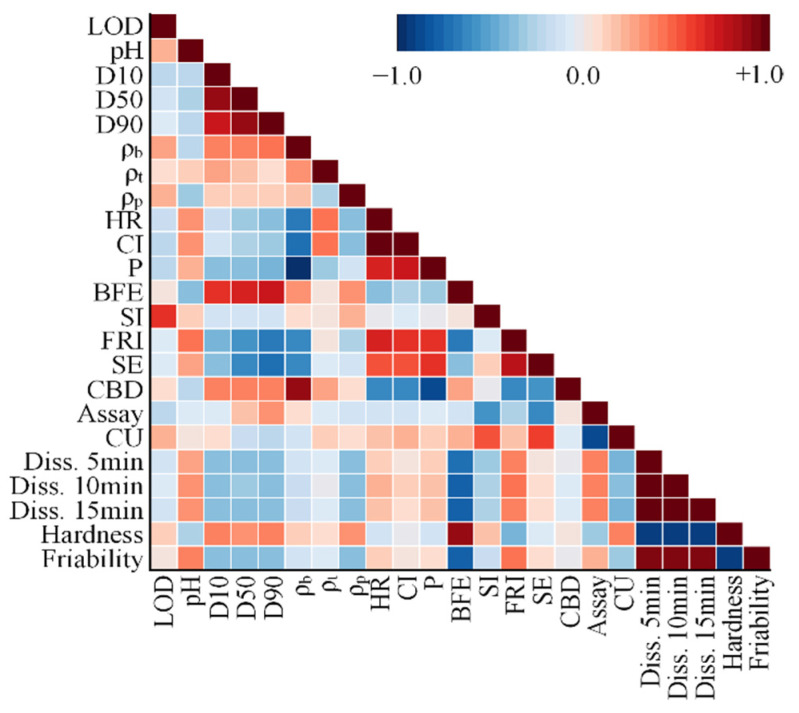
Pearson correlation coefficient matrix. (LOD, loss on drying; ρ_b_, bulk density; ρ_t_, tapped density; ρ_p_, true density; HR, Hausner ratio; CI, compressibility index; P, powder porosity; BFE, basic flowability energy; SI, stability index; FRI, flow rate index; SE, specific energy; CBD, conditioned bulk density; CU, content uniformity; Diss., dissolution).

To identify the impact of MCC variability on the design space, DoE was performed using different MCC manufacturers and grades. As presented in Figure 2d, there was a negative association between A3 and C4 of the same MCC grade in PC4. The effect of the manufacturer change on the design space was thus confirmed using A3 (Pharmacel^®^ 112, DFE Pharma) and C4 (MICROCEL^®^ MC 112, Blanver). As presented in Figure 4a,b, the design spaces of A3 and C4 demonstrated significant differences compared with those of the formulation development. In A3, the design space could not be detected, the yellow region could be observed in C4, but the yellow and dark yellow regions in C4 were smaller than the design space of the formulation development. In addition, the optimal setting for formulation development was not included in the design space. These design space variabilities could possibly be the result of variability in the physicochemical property. As presented in Figure 2b,d, a negative correlation between A3 and C4 was detected in PC4, while PC4 was predominantly affected by SI, LOD, and CBD. C4, which had a positive score in PC4, exhibited high SI and LOD and low CBD (Table 2). In contrast, A3, which exhibited a negative score in PC4, featured low SI and LOD and high CBD (Table 2). These variations might trigger CQA variability. The correlation between the physicochemical properties of MCC and CQAs was based on the use of PCCs, which clarified how the design space variability of A3 and C4 occurred. Based on the PCCs presented in Figure 5 and Table S7, LOD exhibited a negative correlation with dissolution, whereas CBD displayed negative correlations with dissolution and friability. Moreover, SI showed a negative correlation with the assay, dissolution, and friability and a positive correlation with CU. The assay and CU of A3 ranged from 101.91% to 109.64% and from 0.02% to 0.56%, respectively, and those of C4 ranged from 99.43% to 102.67% and from 0.26% to 7.10%, respectively. As A3 had lower SI than C4, the assay of A3 was higher than that of C4, and the CU of A3 was lower than that of C4. The friabilities of A3 and C4 were 0.06–1.78% and 0.05–1.58%, respectively. This could be a result of C4 having higher SI than A3. For A3, dissolution at 5, 10, and 15 min was 9.72–99.34%, 33.52–99.81%, and 38.12–104.36%, respectively, whereas that for C4 was 10.26–104.86%, 35.38–105.36%, and 40.24–110.16%, respectively. This finding demonstrated that A3 had a slower dissolution profile than C4, possibly because A3 exhibited higher CBD than C4.

#### 3.2.4. Effect of Changes in the Grade on Design Space

**Figure 6:**
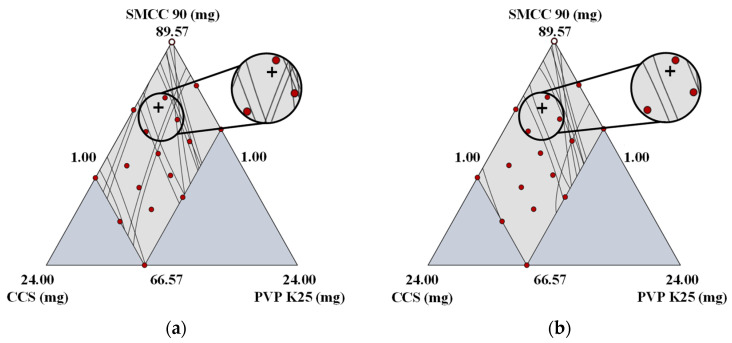
Design space variability triggered by modifications of the grade; (**a**) Design space using D13; (**b**) Design space using D15. The crosshair indicates the optimal setting of formulation development. (SMCC, silicified microcrystalline cellulose; CCS, croscarmellose sodium; PVP, polyvinylpyrrolidone).

The effect of changes in the grade of MCC on the design space was confirmed using D13 (VIVAPUR^®^ 200, JRS Pharma GmbH & Co. KG) and D15 (VIVAPUR^®^ 301, JRS Pharma GmbH & Co. KG). Figure 6a,b presents the design space variability triggered by changes in the grade. Significant differences in the design spaces of D13 and D15 were noticed. In both D13 and D15, the design spaces were not observed, possibly because of the variability in the physicochemical properties triggered by a modification of the grade. As presented in Figure 2a,c, a negative correlation was identified between D13 and D15 in PC2, and PC2 was predominantly affected by LOD, SI, HR, CI, tapped density, and PSD (D10, D50, and D90). D15, which had a positive score in PC2, exhibited high LOD and SI and low HR, CI, tapped density, and PSD (Table 2). In contrast, D13, which had a negative score in PC2, exhibited low LOD and SI and high HR, CI, tapped density, and PSD (Table 2). The variations in physicochemical properties could trigger the variability of CQAs that leads to design space variability. As presented in Figure 5 and Table S7, LOD exhibited a negative correlation with dissolution, whereas PSD had negative correlations with dissolution and friability and a positive correlation with hardness. Moreover, HR and CI exhibited positive correlations with dissolution. Dissolution at 5, 10, and 15 min of D13 was 9.61–100.22%, 34.14–101.15%, and 38.69–103.47%, respectively, and that of D15 was 10.04–104.68%, 35.65–105.65%, and 40.41–108.07%, respectively. Despite the fact that LOD, HR, and CI showed a correlation with dissolution, dissolution was primarily affected by PSD. PSD exhibited a robust negative correlation with dissolution and D13, which displayed high PSD and had slower dissolution profiles than D15. The friabilities of D13 and D15 were 0.03–0.84% and 0.10–2.97%, respectively. PSD had a negative correlation with friability and because D15 had lower PSD than D13, it exhibited high friability. The hardness values of D13 and D15 ranged from 9.31 kp to 11.93 kp and between 4.62 kp and 5.92 kp, respectively. Based on the data in Figure 5 and Table S7, a strong positive correlation between PSD (D10, D50, and D90) and hardness was observed. Consequently, D13, which displayed high PSD, exhibited greater hardness than D15. Of note, there was no significant correlation between tapped density and CQAs, whereas SI had a negative correlation with the assay and a positive correlation with CU. The assay and CU of D13 ranged from 97.71% to 105.13% and between 0.04% and 1.11%, respectively, whereas those of D15 were between 95.24% and 101.44% and 0.30% and 8.35%, respectively. As previously mentioned, SI exhibited a positive correlation with CU and a negative correlation with the assay. D13, which had low SI, exhibited a higher assay and lower CU than D15.

### 3.3. Establishment of a Dissolution Prediction Model Based on PCA-ANNs

#### 3.3.1. Establishment of the PCA-ANN Model

A PCA was performed using the SPSS statistical software package (IBM SPSS Statistics Version 27, SPSS Inc., Chicago, IL, USA). The physicochemical properties of 36 different MCCs were employed to confirm that the dataset was suitable for the PCA. The Kaiser Meyer–Olkin (KMO) test and Bartlett’s sphericity test were performed, and the physicochemical properties featuring a KMO value higher than 0.5 and a *p*-value lower than 0.05 were considered appropriate for PCA. Furthermore, components with an eigenvalue higher than 1 were considered PCs. The eigenvalues, PC variance contribution rates, and cumulative contribution rates are presented in Table S8, whereas the PC factor load and coefficient matrix are presented in Table S9. Based on the data presented in Table S8, four PCs were extracted, and the cumulative contribution rate was 80.122%. Based on the PC coefficient matrix, the PC expression and the corresponding PC score can be acquired. To calculate the scores of the four PCs, each physicochemical property was set as LOD (*a*_1_), pH (*a*_2_), D10 (*a*_3_), D50 (*a*_4_), D90 (*a*_5_), bulk density (*a*_6_), tapped density (*a*_7_), true density (*a*_8_), HR (*a*_9_), CI (*a*_10_), porosity (*a*_11_), BFE (*a*_12_), SI (*a*_13_), FRI (*a*_14_), SE (*a*_15_), and CBD (*a*_16_). The four PCs scores (F1, F2, F3, and F4) were determined using Equations (14)–(17), as presented in Table S10. The four PC scores were selected as the input layer of the ANN model for network training and learning.

F1 = 0.016*a*_1_ − 0.063*a*_2_ + 0.091*a*_3_ + 0.107*a*_4_ + 0.111*a*_5_ + 0.125*a*_6_ + 0.010*a*_7_ + 0.047*a*_8_ − 0.115*a*_9_ − 0.111*a*_10_ − 0.123*a*_11_ + 0.097*a*_12_ − 0.001*a*_13_ − 0.130*a*_14_ − 0.116*a*_15_ + 0.115*a*_16_, (14)

F2 = –0.237*a*_1_ − 0.029*a*_2_ + 0.240*a*_3_ + 0.225*a*_4_ + 0.194*a*_5_ − 0.100*a*_6_ + 0.145*a*_7_ − 0.122*a*_8_ + 0.184*a*_9_ + 0.207*a*_10_ + 0.095*a*_11_ + 0.123*a*_12_ − 0.192*a*_13_ + 0.010*a*_14_ − 0.037*a*_15_ − 0.067*a*_16_, (15)

F3 = 0.164*a*_1_ + 0.217*a*_2_ + 0.005*a*_3_ − 0.029*a*_4_ − 0.056*a*_5_ + 0.208*a*_6_ + 0.436*a*_7_ − 0.250*a*_8_ + 0.144*a*_9_ + 0.116*a*_10_ − 0.220*a*_11_ − 0.142*a*_12_ + 0.069*a*_13_ + 0.062*a*_14_ − 0.039*a*_15_ + 0.184*a*_16_, (16)

F4 = 0.377*a*_1_ + 0.096*a*_2_ + 0.138*a*_3_ + 0.131*a*_4_ + 0.125*a*_5_ − 0.092*a*_6_ + 0.085*a*_7_ + 0.237*a*_8_ + 0.121*a*_9_ + 0.140*a*_10_ + 0.103*a*_11_ + 0.248*a*_12_ + 0.458*a*_13_ − 0.048*a*_14_ + 0.066*a*_15_ − 0.179*a*_16_, (17)

ANNs were created to predict dissolution in formulation development. By selecting four PC scores (F1, F2, F3, and F4) as the input and the dissolution at three different time points (5, 10, and 15 min) as the output, the PCA-ANN model was created between the input and output layers. The data were normalized for standardization, and consequently, the pace of the convergence of the training network while processing the data increased. The scale of the data matrix was set from 0 to 1. Among 36 datasets, 30 were selected as training samples, and the remaining 6 were regarded as validation samples.

**Figure 7:**
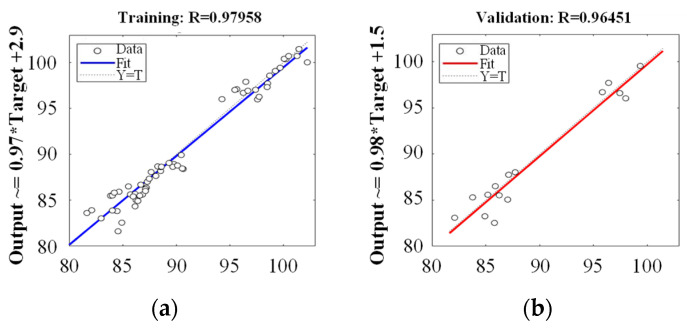

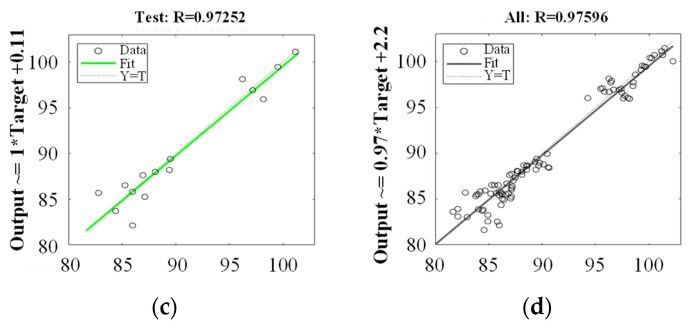
Regression plots; (**a**) Training; (**b**) Validation; (**c**) Test; (**d**) All data set.

**Figure 8:**
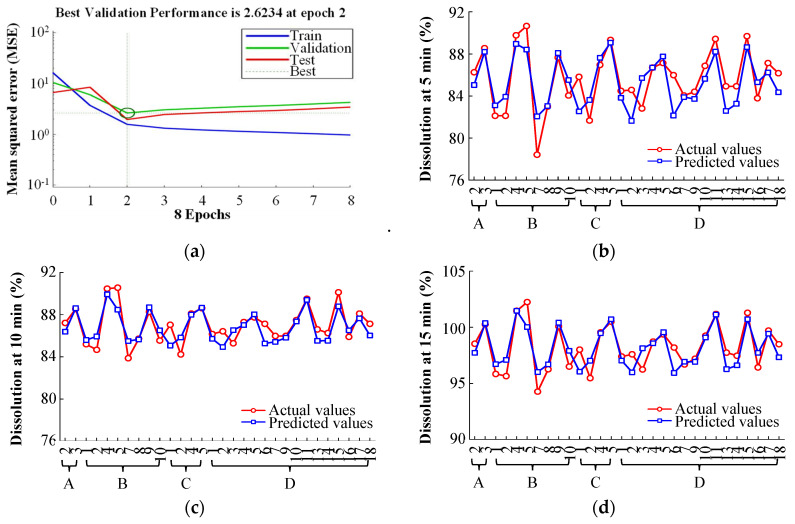
Training and fitting curve; (**a**) Performance of neural network during training; (**b**) Actual vs. Predicted dissolution at 5 min; (**c**) Actual vs. Predicted dissolution at 10 min; (**d**) Actual vs. Predicted dissolution at 15 min. For the reader’s clarity, the MCC abbreviations (X-axis) in (**b**–**d**) have been defined. The manufacturers of MCC are labeled using uppercase letters: A, DFE Pharma; B, FMC BioPolymer; C, Blanver; D, JRS Pharma GmbH & Co. KG. The details of the abbreviations are shown in Table 2.

The predictive performance of PCA-ANNs was evaluated by employing MSE and *R*^2^. The regression analysis obtained from the neural network training tool is presented in Figure 7. The four regression outcomes are presented (training, validation, test, and all). As presented in Figure 7, all regression results revealed *R*^2^ values exceeding 0.9, indicating a good fit between the network and the data. Figure 8a presents the MSE and validation performance of the network. The optimal validation performance was 2.6234 at epoch 2 after six error repetitions, and the process ended at epoch 8. The PCA-ANN model training and fitting curve of dissolution at 5, 10, and 15 min are presented in Figure 8b–d. These results revealed that the developed PCA-ANN model was reliable, and it could be employed as an effective predictive model for dissolution in formulation development.

#### 3.3.2. Model Verification

**Figure 9:**
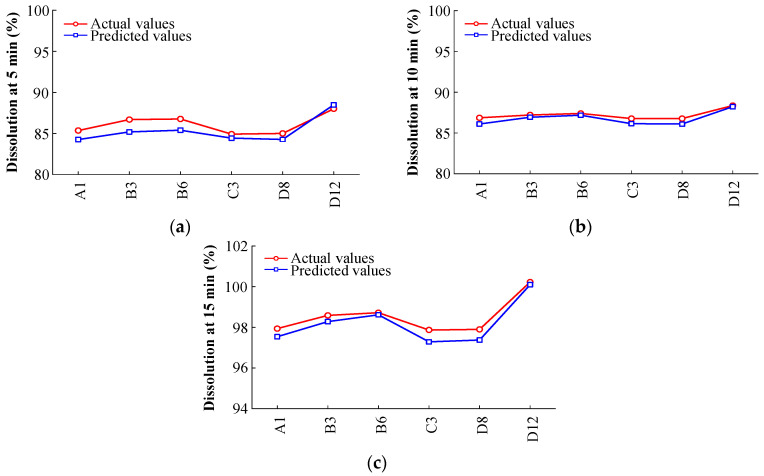
Prediction model verification and fitting curve; (**a**) Actual vs. Predicted dissolution at 5 min; (**b**) Actual vs. Predicted dissolution at 10 min; (**c**) Actual vs. Predicted dissolution at 15 min.

To verify the PCA-ANN model, the validation sample was inserted into the PCA-ANN model. The comparison among actual and predicted values of dissolution at 5, 10, and 15 min are presented in Table S11 and Figure 9. As shown in Figure 9, although the PCA-ANN model tends to slightly underestimate the dissolution profiles, it presented satisfactory results for dissolution at three time points. The absolute error (AE) corresponds to the difference between the actual and predicted values, and the relative error (RE) corresponds to the AE divided by the actual value. As shown in Table S11, the AE and RE ranges of the PCA-ANN model were 0.10–1.50% and 0.10–1.73%, respectively. These results revealed that the PCA-ANN model has decent predictive accuracy for dissolution.

## 4. Conclusions

In the present study, the formulation of an immediate-release tablet containing amlodipine besylate was developed using the QbD approach, and a robust design space was obtained. Of note, the impact of MCC variability on the design space was validated by employing various MCC manufacturers and grades. The 36 different MCCs were associated with variability in physicochemical properties that led to design space variability. After calculating PCCs, the basis of the design space variability was confirmed according to the correlation between the physicochemical properties of MCC and CQAs. The physicochemical properties of MCC, such as LOD, SI, HR, CI, tapped density, PSD, and CBD, mainly caused design space variability. However, these properties are not contained in the specification of pharmacopeia monographs and manufacturers, or the criteria are not harmonized. To ensure the robustness of the drug product quality, the cause of quality variability and the harmonization of excipient specifications need to be understood. A PCA-ANN model was established for predicting dissolution. Using this model, the variability of dissolution could be reduced when MCC variability was noted. The developed PCA-ANN model could accurately predict dissolution at 5, 10, and 15 min with low AE and RE. This study demonstrated that excipient variability leading to variability in drug product quality should be controlled rigorously to ensure a reliable quality of drug products in the QbD approach. Furthermore, it was established that statistical analysis can be utilized to improve understanding of the complex pharmaceutical variables, and the cause of variability can thus be found. Moreover, it was demonstrated that the PCA-ANN model can be used to control variability by predicting drug product quality. Based on these results, the consistent production of high-quality drug products is possible by identifying and controlling the causes of variability in the drug product quality.

## Figures and Tables

**Table 1:** The result of the experiment design and summary of the coded equation and statistical analysis. (*x*_1_, silicified microcrystalline cellulose 90; *x*_2_, croscarmellose sodium; *x*_3_, polyvinylpyrrolidone K25; *y*_1_, hardness; *y*_2_, friability; *y*_3_, dissolution at 5 min; *y*_4_, dissolution at 10 min; *y*_5_, dissolution at 15 min; *y*_6_, assay; *y*_7_, content uniformity).

Run Order	Control Factors			Response Factors						
	*x*_1_ (mg)	*x*_2_ (mg)	*x*_3_ (mg)	*y*_1_ (kp)	*y*_2_ (%)	*y*_3_ (%)	*y*_4_ (%)	*y*_5_ (%)	*y*_6_ (%)	*y*_7_ (%)
1	85.07	1.00	5.50	9.68	0.06	30.92	56.80	70.76	100.6	1.51
2	78.07	8.00	5.50	9.35	0.10	94.27	92.18	96.26	103.9	2.48
3	81.57	4.50	5.50	9.58	0.08	74.28	80.83	85.95	102.4	0.58
4	75.57	15.00	1.00	7.96	0.31	89.38	96.50	84.08	99.9	0.12
5	80.57	1.00	10.00	10.20	0.01	9.37	33.29	37.73	99.7	0.38
6	79.32	4.50	7.75	9.88	0.04	70.43	75.19	77.70	99.8	2.51
7	71.07	15.00	5.50	9.10	0.15	98.05	99.11	92.15	97.9	1.87
8	66.57	15.00	10.00	8.86	0.05	93.36	94.92	94.27	98.9	0.81
9	76.82	11.50	3.25	8.68	0.14	93.08	93.33	92.96	100.6	0.28
10	73.57	8.00	10.00	9.84	0.02	95.55	96.72	95.82	100.4	1.15
11	75.82	8.00	7.75	9.65	0.05	94.56	88.39	91.54	98.0	0.86
12	82.57	8.00	1.00	8.37	0.28	93.34	95.69	100.06	96.6	0.17
13	83.82	4.50	3.25	9.12	0.11	74.28	86.47	98.71	103.1	0.05
14	74.57	11.50	5.50	9.24	0.13	97.73	98.64	98.51	99.6	1.59
15	89.57	1.00	1.00	8.92	0.24	34.63	65.68	83.13	100.9	2.12
16	80.32	8.00	3.25	8.93	0.13	95.62	96.83	100.90	98.4	1.42
17	72.32	11.50	7.75	9.64	0.07	100.63	94.63	96.19	98.3	0.09
**Response factors**	**Main effects of control factors**			**Mutual interactions between control factors**			**Statistical analysis of the coded equation**			
	***x*** **_1_**	***x*** **_2_**	***x*** **_3_**	***x*** **_1_** ***x*** **_2_**	***x*** **_1_** ***x*** **_3_**	***x*** **_2_** ***x*** **_3_**	***p*** **-value**	***R* ^2^**	**Adjusted** ***R* ^2^**	**Predicted *R*^2^**
*y*_1_	8.75	7.52	6.51	-	9.76	8.50	<0.0001	0.98	0.97	0.92
*y*_2_	0.23	0.42	0.52	−0.15	−1.38	−1.66	<0.0001	0.95	0.93	0.88
*y*_3_	36.81	−30.24	−13.87	378.99	-	505.78	<0.0001	0.98	0.97	0.91
*y*_4_	66.60	28.09	−2.61	207.79	-	342.00	<0.0001	0.95	0.93	0.84
*y*_5_	86.55	−4.84	−23.92	213.72	-	457.32	<0.0001	0.94	0.93	0.81

**Table 2:** Physicochemical properties of 36 different MCCs. (LOD, loss on drying; HR, Hausner ratio; CI, compressibility index; BFE, basic flowability energy; SI, stability index; FRI, flow rate index; SE, specific energy; CBD, conditioned bulk density.)

Manufacturer	Brand Name	Abbreviation	LOD	pH	D10	D50	D90	Bulk Density	Tapped Density	True Density	HR	CI	Powder Porosity	BFE	SI	FRI	SE	CBD
			(%)		(µm)	(µm)	(µm)	(g/mL)	(g/mL)	(g/mL)		(%)	(%)	(mJ)			(mJ/g)	(g/mL)
DFE Pharma	Pharmacel^®^ 101	A1	3.0	6.1	22.00	62.20	147.0	0.31	0.43	0.800	1.39	27.91	61.25	281.0	1.32	1.53	9.420	0.332
	Pharmacel^®^ 102	A2	3.4	6.0	43.00	123.4	248.0	0.31	0.42	0.802	1.35	26.19	61.35	278.0	1.30	1.43	5.920	0.342
	Pharmacel^®^ 112	A3	1.1	5.7	30.00	90.00	186.0	0.33	0.47	0.791	1.42	29.79	58.28	189.0	1.00	1.33	5.950	0.379
FMC BioPolymer	Avicel^®^ PH-101	B1	3.2	6.1	21.40	61.70	154.0	0.30	0.51	0.802	1.70	41.18	62.59	311.0	1.32	1.72	9.720	0.322
	Avicel^®^ PH-102	B2	3.6	6.2	38.20	135.0	273.6	0.32	0.51	0.802	1.59	37.25	60.08	298.0	1.35	1.38	6.150	0.352
	Avicel^®^ PH-103	B3	2.6	6.3	28.20	66.30	162.8	0.28	0.49	0.793	1.75	42.86	64.69	258.0	1.15	2.13	8.920	0.380
	Avicel^®^ PH-105	B4	2.8	6.5	9.000	28.00	62.00	0.24	0.60	0.791	2.50	60.00	69.66	38.1	1.14	3.01	10.20	0.245
	Avicel^®^ PH-112	B5	1.3	6.2	24.00	143.0	284.0	0.30	0.54	0.792	1.80	44.44	62.12	198.0	1.00	1.29	6.100	0.349
	Avicel^®^ PH-113	B6	1.2	5.9	24.00	68.00	154.0	0.28	0.55	0.803	1.96	49.09	65.13	245.0	1.03	2.05	8.710	0.380
	Avicel^®^ PH-200	B7	2.8	5.9	114.4	248.6	400.7	0.32	0.55	0.800	1.72	41.82	60.00	395.0	1.24	1.29	7.420	0.359
	Avicel^®^ PH-200LM	B8	1.1	6.0	168.0	247.0	439.0	0.34	0.56	0.800	1.65	39.29	57.50	392.0	1.13	1.32	7.320	0.379
	Avicel^®^ PH-301	B9	3.2	6.1	48.20	53.60	148.7	0.40	0.59	0.805	1.48	32.20	50.31	204.0	1.11	1.52	7.420	0.430
	Avicel^®^ PH-302	B10	3.1	6.3	57.80	139.4	242.3	0.42	0.59	0.795	1.40	28.81	47.17	298.0	1.14	1.25	6.120	0.420
Blanver	MICROCEL^®^ MC 12	C1	3.1	6.1	42.10	160.0	367.8	0.37	0.49	0.802	1.32	24.49	53.87	361.0	1.01	1.32	5.410	0.376
	MICROCEL^®^ MC 101	C2	2.8	6.3	26.50	71.10	151.8	0.30	0.46	0.801	1.53	34.78	62.53	302.0	1.21	1.66	9.680	0.322
	MICROCEL^®^ MC 102	C3	2.1	6.2	33.80	94.60	234.0	0.32	0.51	0.802	1.59	37.25	60.08	293.0	1.00	1.30	6.310	0.352
	MICROCEL^®^ MC 112	C4	3.2	6.9	27.10	102.5	245.1	0.32	0.48	0.803	1.50	33.33	60.17	221.0	1.30	1.42	7.210	0.369
	MICROCEL^®^ MC 200	C5	3.1	5.8	73.00	180.0	264.0	0.35	0.47	0.801	1.34	25.53	56.31	201.0	1.17	1.47	6.070	0.418
JRS Pharma GmbH & Co. KG	PROSOLV^®^ SMCC 50	D1	2.8	5.6	25.00	65.00	162.4	0.33	0.44	0.809	1.33	25.00	59.22	300.0	1.13	1.59	9.250	0.352
	PROSOLV^®^ SMCC 50 LD	D2	1.3	5.7	21.00	56.30	156.2	0.24	0.45	0.798	1.88	46.67	69.93	321.0	1.05	1.72	9.570	0.262
	PROSOLV^®^ SMCC 90	D3	1.2	5.4	42.30	142.7	251.0	0.35	0.43	0.813	1.23	18.60	56.92	271.0	1.21	1.18	6.320	0.382
	PROSOLV^®^ SMCC HD 90	D4	2.1	5.8	54.20	118.5	243.0	0.42	0.53	0.798	1.26	20.75	47.37	274.0	1.05	1.21	6.410	0.452
	PROSOLV^®^ SMCC 90 LM	D5	2.2	5.7	46.80	125.0	251.3	0.30	0.44	0.806	1.47	31.82	62.79	219.0	1.02	1.63	6.120	0.368
	VIVAPUR^®^ 12	D6	3.1	6.1	67.20	198.8	420.0	0.33	0.46	0.800	1.39	28.26	58.76	388.0	1.02	1.28	5.570	0.336
	VIVAPUR^®^ 14	D7	1.0	6.0	78.10	170.0	428.1	0.36	0.48	0.798	1.33	25.00	54.91	332.0	0.92	1.39	6.710	0.400
	VIVAPUR^®^ 101	D8	2.3	5.7	26.20	75.30	167.2	0.31	0.45	0.800	1.45	31.11	61.25	289.0	1.19	1.61	9.510	0.332
	VIVAPUR^®^ 102	D9	2.1	5.6	34.60	103.2	252.2	0.31	0.50	0.800	1.61	38.00	61.25	301.0	1.10	1.33	6.560	0.342
	VIVAPUR^®^ 103	D10	1.1	6.3	29.10	65.00	123.0	0.28	0.44	0.792	1.57	36.36	64.63	226.0	0.99	1.85	8.730	0.380
	VIVAPUR^®^ 105	D11	1.2	6.5	8.000	26.00	32.00	0.24	0.45	0.795	1.88	46.67	69.80	41.9	1.01	3.37	11.40	0.245
	VIVAPUR^®^ 112	D12	1.3	6.2	38.00	147.8	294.1	0.33	0.45	0.791	1.36	26.67	58.28	204.0	1.10	1.32	6.900	0.379
	VIVAPUR^®^ 200	D13	1.5	5.9	138.0	250.0	325.0	0.32	0.48	0.802	1.50	33.33	60.11	380.0	1.04	1.40	7.010	0.359
	VIVAPUR^®^ XLM200	D14	1.3	5.9	127.0	252.0	337.0	0.36	0.51	0.801	1.42	29.41	55.06	372.0	1.01	1.32	6.920	0.399
	VIVAPUR^®^ 301	D15	3.6	6.1	28.50	78.10	177.3	0.40	0.46	0.800	1.15	13.04	50.00	185.0	1.27	1.48	7.230	0.430
	VIVAPUR^®^ 302	D16	2.6	5.8	47.90	130.0	187.0	0.39	0.55	0.796	1.41	29.09	51.00	304.0	1.05	1.39	6.580	0.441
	Heweten^®^ 101	D17	2.2	6.5	25.00	67.10	151.5	0.28	0.40	0.805	1.43	30.00	65.22	208.0	1.00	1.82	9.400	0.332
	Heweten^®^ 102	D18	2.4	6.1	34.50	109.4	271.1	0.29	0.41	0.802	1.41	29.27	63.84	281.0	1.00	1.40	6.120	0.322

## Data Availability

The authors confirm that the data supporting the findings of this study are available within the article and its Supplementary Materials.

## Supplementary Materials

The following supporting information can be downloaded at: https://www.mdpi.com/article/10.3390/pharmaceutics14112416/s1, Table S1: Experimental design and target value for formulation optimization. (SMCC, silicified microcrystalline cellulose; CCS, croscarmellose sodium; PVP, polyvinylpyrrolidones); Table S2: Quality target product profile and critical quality attributes for amlodipine tablet. (QTPP, quality target product profile; CQA, critical quality attribute); Table S3: Initial risk assessment for formulation development. (MAs, material attributes; CQAs, critical quality attributes; RPN, risk priority number; S, severity; P, probability of occurrence; D, detectability; CU, content uniformity; SMCC, silicified microcrystalline cellulose; CCS, croscarmellose sodium; PVP, polyvinylpyrrolidones; St-Mg, magnesium stearate); Table S4: Result of design space validation. (SMCC, silicified microcrystalline cellulose; CCS, croscarmellose sodium; PVP, polyvinylpyrrolidone); Table S5: Comparison of the MCC monograph specification in pharmacopeia and manufacturers. (MCC, microcrystalline cellulose; USP–NF, United States Pharmacopeia–National Formulary; Ph. Eur., European Pharmacopoeia; JP, Japanese Pharmacopeia; KP, Korean Pharmacopeia; NMT, not more than); Table S6: Risk assessment for microcrystalline cellulose. (CQAs, critical quality attributes; CU, content uniformity; IR, Infrared spectroscopy; LOD, loss on drying; PSD, particle size distribution); Table S7: Pearson correlation coefficients. (LOD, loss on drying; ρ_p_, true density; ρ_b_, bulk density; ρ_t_, tapped density; HR, Hausner ratio; CI, compressibility index; P, powder porosity; BFE, basic flowability energy; SI, stability index; FRI, flow rate index; SE, specific energy; CBD, conditioned bulk density; CU, content uniformity; Diss., dissolution); Table S8: Explanation of variance; Table S9: Component and score coefficient matrix. (LOD, loss on drying; BFE, basic flowability energy; SI, stability index; FRI, flow rate index; SE, specific energy; CBD, conditioned bulk density); Table S10: Principal component scores and CQAs. (F1–F4 are four PCs scores); Table S11: Comparison of the actual and predicted values of dissolution in the PCA-ANN model. (AE, absolute error; RE, relative error).

## Abbreviations

APIs, active pharmaceutical ingredients; PSD, particle size distribution; MCC, microcrystalline cellulose; QbD, quality by design; CMAs, critical material attributes; CQAs, critical quality attributes; DoE, design of experiment; ANN, artificial neural network; SMCC, silicified microcrystalline cellulose; PVP, polyvinylpyrrolidone; CCS, croscarmellose sodium; St-Mg, Magnesium stearate; HPLC, high-performance liquid chromatography; RPN, risk priority number; MA, material attribute; CU, content uniformity; R^2^, coefficient of determination; ANOVA, analysis of variance; USP–NF, United States Pharmacopeia–National Formulary; CoA, certificate of analysis; LOD, loss on drying; HR, Hausner ratio; CI, compressibility index; BFE, basic flowability energy; SI, stability index; FRI, flow rate index; SE, specific energy; CBD, conditioned bulk density; PCA, principal component analysis; PCC, Pearson correlation coefficient; PCs, principal components; MSE, mean square error; QTPP, quality target product profile; PDG, Pharmacopeial Discussion Group; Ph. Eur., European Pharmacopoeia; JP, Japanese Pharmacopeia; KMO, Kaiser Meyer–Olkin; AE, absolute error; RE, relative error.
