# Supplementary material for: Control Strategy for Excipient Variability in the Quality by Design Approach Using Statistical Analysis and Predictive Model: Effect of Microcrystalline Cellulose Variability on Design Space
Source: Pharmaceutics. 2022 Nov 8;14(11):2416. doi: 10.3390/pharmaceutics14112416 (PMC9696966; doi:10.3390/pharmaceutics14112416)
Supplement: Supplementary file 1 [file pharmaceutics-14-02416-s001.zip › pharmaceutics-2002529-supplementary.pdf]

pharmaceutics

Supplementary Material

Control Strategy for Excipient Variability in the Quality by Design Approach using Statistical Analysis and Predictive Model: Effect of Microcrystalline Cellulose Variability on Design Space

Ji Yeon Kim[1], Du Hyung Choi[1,*]

Table S1. Experimental design and target value for formulation optimization. (SMCC, silicified microcrystalline cellulose; CCS, croscarmellose sodium; PVP, polyvinylpyrrolidones).

Control Factors (x)		Level	
		Less Than	Less Than
x_1	SMCC 90 (mg)	66.6	89.6
x_2	CCS (mg)	1.0	15.0
x_3	PVP K25 (mg)	1.0	10.0
**Response factors (y)**		**Standard**	**Target**
y_1	Hardness (kp)	-	7–10
y_2	Friability (%)	Under 1.0	Under 0.3
y_3	Dissolution 5 min (%)	-	66.3–96.3
y_4	Dissolution 10min (%)	-	71.7–100.0
y_5	Dissolution 15min (%)	Upper 85	Upper 85
y_6	Assay (%)	90.0–110.0	95.0–105.0
y_7	Content uniformity (%)	Less than 15.0	Less than 5.0

Table S2. Quality target product profile and critical quality attributes for amlodipine tablet. (QTPP, quality target product profile; CQA, critical quality attribute).

QTPP elements		Target	Justification	Is this a CQA?
Dosage form		Tablet	Pharmaceutical equivalence requirement: same dosage form	No
Dosage design		Immediate release tablet containing 6.94 mg of amlodipine besylate	Immediate release design needed to meet label claims	No
Route of administration		Oral	Pharmaceutical equivalence requirement: same route of administration	No
Dosage strength		Amlodipine besylate 6.94 mg	Pharmaceutical equivalence requirement: same strength	No
Pharmacokinetic properties		Bioequivalent to the Reference drug	Bioequivalence requirement	No
Stability		At least 24 months shelf-life at room temperature	Equivalent to or better than Reference drug shelf-life	No
Drug product	Assay	95~105 %	Variability in the content will affect safety and efficacy. Both formulation and process variables	Yes

quality attributes			impact content, so this CQA should be evaluated throughout the product and process development.	
	Content uniformity	Conforms to USP<905> uniformity of dosage units	Variability in content uniformity will affect safety and efficacy. Both formulation and process variables impact content uniformity, so this CQA should be evaluated throughout product and process development.	Yes
	Dissolution	Similar to the Reference drug	Failure to meet the dissolution specification can impact bioavailability. Both formulation and process variables affect the dissolution profile. This CQA will be investigated throughout the formulation and process development.	Yes
	Hardness	Similar to the Reference drug	Hardness should be rigid enough not to break during routine handling and should not significantly affect dissolution	Yes
	Friability	Not more than 0.3 % w/w	Friability is necessary to minimize the impact of drug loss on the qualitative and efficacy of the drug and to maintain the formulation during packaging.	Yes
Identification		237 nm	Easy to confirm to show unique absorption wavelength in a specific range	No
Degradation products		Pharmaceutical equivalence requirement: Must meet the same applicable (quality) standards (i.e., identity, assay, purity, and quality).		No
Residual solvents				No
Microbial limits				No
Container closure system		Tight and light-resistant containers	Container closure system qualified as suitable for this drug product and needed to achieve the target shelf-life and to ensure tablet integrity during shipping	No

Table S3. Initial risk assessment for formulation development. (MAs, material attributes; CQAs, critical quality attributes; RPN, risk priority number; S, severity; P, probability of occurrence; D, detectability; CU, content uniformity; SMCC, silicified microcrystalline cellulose; CCS, croscarmellose sodium; PVP, polyvinylpyrrolidones; St-Mg, magnesium stearate).

**MAs**	**CQAs**	***S***	***P***	***D***	**RPN**	**Risk Degree**	**Justification**
SMCC 90	Assay	3	3	4	36	Medium	SMCC is considered to have an effect on assay and CU because it accounts for a high ratio in the tablet. Therefore, SMCC poses a medium risk to the assay and CU.
	CU	3	3	4	36	Medium	
	Dissolution	4	3	4	48	High	The porosity of SMCC accelerates the swelling and disintegration of tablets, so it can have a great influence on dissolution. Therefore, SMCC poses a high risk to the dissolution.
	Hardness	4	4	3	48	High	SMCC used as a filler has good compressibility. Therefore, the hardness and friability depending on the amount used, so SMCC poses a high risk to the hardness.
	Friability	4	4	3	48	High	
CCS	Assay	2	1	4	8	Low	CCS is considered to have a negligible effect on the assay and CU it accounts for a low ratio in the
	CU	2	1	4	8	Low	

							tablet. Therefore, CCS poses a low risk to the assay and CU.
	Dissolution	5	5	4	100	High	CCS used as disintegrant has properties of absorbing water and swelling it can affect dissolution. Therefore, CCS poses a high risk to dissolution.
	Hardness	2	3	3	18	Low	CCS is considered to have a negligible effect on the hardness and friability it accounts for a low ratio in the tablet. Therefore, CCS poses a low risk to the hardness and friability.
	Friability	2	3	3	18	Low	
PVP K25	Assay	2	1	4	8	Low	PVP is considered to have a negligible effect on the assay and CU it accounts for a low ratio in the tablet. Therefore, PVP poses a low risk to the assay and CU.
	CU	2	1	4	8	Low	
	Dissolution	5	5	4	100	High	PVP was used as a binder directly related to binding force, it was classified to high impact on dissolution, hardness, and friability.
	Hardness	4	4	3	48	High	
	Friability	4	4	3	48	High	
St-Mg	Assay	2	1	4	8	Low	Magnesium stearate is considered to have a negligible effect on the assay it accounts for a low ratio in the tablet. Therefore, magnesium stearate poses a low risk to the assay.
	CU	2	2	4	16	Low	Long lubrication times can affect CU due to particle delamination, but magnesium stearate is used in a low proportion in tablets and therefore has less effect on CU. Therefore, magnesium stearate poses a low risk to the CU.
	Dissolution	2	1	4	8	Low	Magnesium stearate is considered to have a negligible effect on the dissolution, hardness, and friability since it accounts for a low ratio in the tablet. Therefore, magnesium stearate poses a low risk to dissolution, hardness, and friability.
	Hardness	2	1	3	6	Low	
	Friability	2	1	3	6	Low	

Criteria of severity, probability of occurrence, and detectability

**Score**	**Severity (S)**	**Probability of occurrence (P)**	**Detectability (D)**
1	Irrelevant	An unlikely probability of occurrence	High degree of detectability
2	Slight	A remote probability of occurrence	Good detectability
3	Important	An occasional probability of occurrence	Likely to detect
4	Critical	A moderate probability of occurrence	Fair detectability
5	Disastrous	A high probability of occurrence	Low or no detectability

Criteria of RPN

**Risk Degree**	**RPN**	**Justification**
Low	1-19	The management is not required since it has little effect on CQAs.

Medium	20-39	The management is necessary since it has a medium effect on CQAs.
High	40-125	The management must be necessary since it has a high effect on CQAs if occurs the change of the condition.

Table S4. Result of design space validation. (SMCC, silicified microcrystalline cellulose; CCS, croscarmellose sodium; PVP, polyvinylpyrrolidone).

**Optimal settings**			**Response factors**				
x_1	x_2	x_3	y_1	y_2	y_3	y_4	y_5
**SMCC 90 (mg)**	**CCS (mg)**	**PVP K25 (mg)**	**Hardness (kp)**	**Friability (%)**	**Dissolution (%)**		
					**5 min**	**10 min**	**15 min**
82.30	5.80	3.47	9.08	0.14	82.81	88.71	96.24
Target value			9.40	0.13	81.30	86.70	90.10
Absolute bias			0.32	0.01	1.51	2.01	6.14
Relative bias (%)			3.40	7.69	1.86	2.32	6.81

Table S5. Comparison of the MCC monograph specification in pharmacopeia and manufacturers. (MCC, microcrystalline cellulose; USP–NF, United States Pharmacopeia–National Formulary; Ph. Eur., European Pharmacopoeia; JP, Japanese Pharmacopeia; KP, Korean Pharmacopeia; NMT, not more than).

**Attributes**	**Pharmacopeia**				**Manufacturer**				
	**USP–NF Monograph**	**Ph. Eur. Monograph**	**JP Monograph**	**KP Monograph**	**DFE Pharma**	**FMC BioPolymer**	**Blanver**	**JRS Pharma GmbH & Co. KG**	
								**VIVAPUR®/ Heweten®**	**PROSOLV® SMCC**
Definition	○	○	○	○	×	×	×	×	×
Characters-Appearance	×	○	×	×	×	×	○	×	×
Characters-Solubility	×	○	×	×	×	×	○	×	○
Description	×	×	○ (Appearance, solubility)	○ (Appearance, solubility)	×	×	×	○ (Appearance, solubility)	×
Identification[*]	○								
Residue on ignition (Sulfated ash)[*, a]	NMT 0.1%	NMT 0.1%	NMT 0.1%	NMT 0.1%	NMT 0.05%	NMT 0.05%	NMT 0.05%	NMT 0.05%	1.8-2.2%
Residual solvent	×	×	×	×	×	×	×	×	○
Solubility (Ammoniacal solution of copper tetramine R)	×	○	×	×	○	○	×	○	×
Conductivity[*, a]	75 µS/cm	75 µS/cm	75 µS/cm	75 µS/cm	75 µS/cm	75 µS/cm	75 µS/cm	50 µS/cm	75 µS/cm
pH[*, a]	5.0–7.5	5.0–7.5	5.0–7.5	5.0–7.5	5.5–7.0	5.5–7.0	5.0–7.0	5.0–7.0	5.0–7.0
Loss on drying[*, a]	NMT 7.0%	NMT 7.0%	NMT 7.0%	NMT 7.0%	○ (Dependent on product grade)	○ (Dependent on product grade)	○ (Dependent on product grade)	○ (Dependent on product grade)	○ (Dependent on product grade)

Bulk density	○	×	○	○	○ (Dependent on product grade)	○ (Dependent on product grade)	○ (Dependent on product grade)	○ (Dependent on product grade)	○ (Dependent on product grade)
Tapped density	×	×	×	×	×	×	×	×	○
Particle size[c]	×	×	×	×	○ (Dependent on product grade)	○ (Dependent on product grade)	○ (Dependent on product grade)	○ (Dependent on product grade)	○ (Dependent on product grade)
Particle size distribution	○	○	×	×	○ (Dependent on product grade)	○ (Dependent on product grade)	×	○ (Dependent on product grade)	○ (Dependent on product grade)
Powder flow	×	○	×	×	×	×	×	○	×
Water-soluble substances[*, a]	12.5 mg (0.25%)	12.5 mg (0.25%)	12.5 mg	12.5 mg	0.20%	12.5 mg (0.25%)	0.24%	0.24%	0.24%
Ether-soluble substances[*, a]	5.0 mg (0.05%)	5.0 mg (0.05%)	5.0 mg	5.0 mg	0.05%	5.0mg	0.05%	0.05%	0.05%
Heavy metals	×	NMT 10 ppm	NMT 10 ppm	NMT 10 ppm	NMT 10 ppm	NMT 10 ppm	NMT 10 ppm	NMT 10 ppm	NMT 10 ppm
Mercury	×	×	×	○	NMT 1.0 ppm	×	×	×	×
Cadmium	×	×	×	○	NMT 1.0 ppm	×	×	×	×
Lead	×	×	×	○	NMT 2.0 ppm	×	×	×	×
Arsenic	×	×	×	○	NMT 4.0 ppm	NMT 2.0 ppm	×	×	×
Starch	×	×	×	○	○	○	×	×	×
Total aerobic microbial count[*, a]	10^3 CFU/g	10^3 CFU/g	10^3 CFU/g	10^3 CFU/g	100 CFU/g	100 CFU/g	10^3 CFU/g	100 CFU/g	100 CFU/g
Total combined molds and yeasts count[*, a]	10^2 CFU/g	10^2 CFU/g	10^2 CFU/g	10^2 CFU/g	20 CFU/g	20 CFU/g	10^2 CFU/g	20 CFU/g	20 CFU/g

Absence of Staphylococcus aureus[*, b]					◯				
Absence of Pseudomonas aeruginosa[*, b]					◯				
Absence of Escherichia coli[*, b]					◯				
Absence of Salmonella species[*, b]					◯				
Absence of Enterobacteriaceae	×	×	×	×	×	×	×	◯	×
Absence of Coliform species	×	×	×	×	×	◯	×	×	×
Packaging and Storage	Tight containers	×	Tight containers	Tight containers	A white multilayered polyethylene bag with a polyethylene inner liner. Keep in original, unopened packing in ambient conditions, protected from humidity and away from strongly odorous materials	Store at ambient conditions. Keep containers sealed	×	Protect from excessive heat and moisture. Keep container closed	×

**Local requirements**		
**USP–NF Monograph**	**Ph. Eur. Monograph**	**JP Monograph**
Particle size distribution estimation by analytical sieving	Solubility, functionality-related characteristics (particle size distribution, powder flow)	Definition (reference to labeling), heavy metals

[*] Attributes that were harmonized among pharmacopeia.

[a] Attributes that were contained in all pharmacopeia and manufacturers, but the criteria were not harmonized.

[b] Attributes that were contained in all pharmacopeia and manufacturers.

[c] Attributes that were contained in manufacturers.

Table S6. Risk assessment for microcrystalline cellulose. (CQAs, critical quality attributes; CU, content uniformity; IR, Infrared spectroscopy; LOD, loss on drying; PSD, particle size distribution).

**Attributes**	**CQAs**				
	**Assay**	**CU**	**Dissolution**	**Hardness**	**Friability**
Appearance	Low	Low	Low	Low	Low
Solubility	Low	Low	Medium	Low	Low
Identification A (IR)	Low	Low	Low	Low	Low
Identification B (Wet chemistry)	Low	Low	Low	Low	Low
Identification C (Degree of polymerization)	Medium	Medium	Medium	Medium	Medium
Residue on ignition (Sulfated ash)	Low	Low	Low	Low	Low
Residual solvent	Low	Low	Low	Low	Low
Solubility (Ammoniacal solution of copper tetramine R)	Low	Low	Low	Low	Low
Conductivity	Low	Low	Low	Low	Low
pH	Low	Low	Medium	Low	Low
Loss on drying	Medium	Medium	High	High	High
Bulk density	High	High	High	High	High
Tapped density	High	High	High	High	High
True density	Low	Low	Low	Medium	Medium
Particle size	High	High	High	High	High
Particle size distribution	High	High	High	High	High
Powder flow	Medium	Medium	Medium	Medium	Medium
Water-soluble substances	Low	Low	Low	Low	Low
Ether-soluble substances	Low	Low	Low	Low	Low
Heavy metals	Low	Low	Low	Low	Low
Mercury	Low	Low	Low	Low	Low
Cadmium	Low	Low	Low	Low	Low
Lead	Low	Low	Low	Low	Low
Arsenic	Low	Low	Low	Low	Low
Starch	Low	Low	Low	Low	Low
Total aerobic microbial count	Low	Low	Low	Low	Low
Total combined molds and yeasts count	Low	Low	Low	Low	Low
Absence of *Staphylococcus aureus*	Low	Low	Low	Low	Low
Absence of *Pseudomonas aeruginosa*	Low	Low	Low	Low	Low
Absence of *Escherichia coli*	Low	Low	Low	Low	Low

Absence of *Salmonella species*	Low	Low	Low	Low	Low
Absence of *Enterobacteriaceae*	Low	Low	Low	Low	Low
Absence of *Coliform species*	Low	Low	Low	Low	Low
Packaging and Storage	Low	Low	Low	Low	Low

Table S7. Pearson correlation coefficients. (LOD, loss on drying; ϱ_p, true density; ϱ_b, bulk density; ϱ_t, tapped density; HR, Hausner ratio; CI, compressibility index; P, powder porosity; BFE, basic flowability energy; SI, stability index; FRI, flow rate index; SE, specific energy; CBD, conditioned bulk density; CU, content uniformity; Diss., dissolution).

Variable	LOD	pH	D10	D50	D90	ϱ_b	ϱ_t	ϱ_p	HR	CI	P	BFE	SI	FRI	SE	CBD	Assay	CU	Diss. 5min	Diss. 10min	Diss. 15min	Hardness	Friability
**LOD**	1.000																						
**pH**	0.248	1.000																					
**D10**	-0.209	-0.235	1.000																				
**D50**	-0.148	-0.252	0.894	1.000																			
**D90**	-0.086	-0.209	0.743	0.897	1.000																		
**ϱ_b**	0.256	-0.242	0.364	0.396	0.405	1.000																	
**ϱ_t**	0.085	0.107	0.269	0.164	0.097	0.319	1.000																
**ϱ_p**	0.237	-0.337	0.123	0.107	0.143	0.161	-0.275	1.000															
**HR**	-0.167	0.311	-0.191	-0.304	-0.367	-0.692	0.446	-0.382	1.000														
**CI**	-0.202	0.313	-0.143	-0.257	-0.316	-0.719	0.421	-0.371	0.972	1.000													
**P**	-0.247	0.229	-0.361	-0.394	-0.401	-0.999	-0.332	-0.119	0.680	0.707	1.000												
**BFE**	0.037	-0.381	0.618	0.651	0.702	0.328	0.022	0.333	-0.374	-0.267	-0.317	1.000											
**SI**	0.648	0.108	-0.111	-0.110	-0.143	0.051	0.003	0.239	-0.050	-0.072	-0.040	0.030	1.000										
**FRI**	-0.095	0.403	-0.412	-0.577	-0.675	-0.644	0.027	-0.289	0.695	0.626	0.637	-0.687	-0.075	1.000									
**SE**	-0.089	0.274	-0.370	-0.612	-0.703	-0.602	-0.097	-0.109	0.529	0.509	0.601	-0.368	0.149	0.758	1.000								
**CBD**	0.077	-0.247	0.358	0.374	0.353	0.862	0.260	0.095	-0.633	-0.625	-0.863	0.266	-0.045	-0.614	-0.590	1.000							
**Assay**	-0.232	-0.063	-0.070	0.174	0.308	0.068	-0.099	-0.147	-0.144	-0.145	-0.075	-0.105	-0.551	-0.278	-0.611	0.008	1.000						
**CU**	0.213	0.019	0.070	-0.160	-0.227	-0.113	0.145	0.100	0.191	0.224	0.119	0.245	0.503	0.154	0.591	-0.054	-0.855	1.000					
**Diss. 5min**	-0.106	0.276	-0.383	-0.356	-0.361	-0.119	-0.097	-0.371	0.111	0.020	0.105	-0.750	-0.313	0.372	0.013	-0.019	0.388	-0.440	1.000				
**Diss. 10min**	-0.084	0.328	-0.367	-0.347	-0.353	-0.167	-0.034	-0.384	0.209	0.101	0.153	-0.774	-0.259	0.417	0.073	-0.099	0.370	-0.388	0.971	1.000			
**Diss. 15min**	-0.107	0.316	-0.392	-0.369	-0.380	-0.179	-0.098	-0.363	0.163	0.074	0.166	-0.785	-0.276	0.396	0.067	-0.081	0.384	-0.416	0.984	0.987	1.000		
**Hardness**	0.134	-0.261	0.366	0.338	0.394	0.131	0.068	0.326	-0.143	-0.041	-0.119	0.851	0.174	-0.403	-0.070	0.022	-0.308	0.385	-0.905	-0.915	-0.933	1.000	
**Friability**	0.024	0.362	-0.359	-0.363	-0.378	-0.112	-0.096	-0.351	0.116	0.007	0.099	-0.776	-0.159	0.427	0.092	-0.008	0.249	-0.305	0.919	0.940	0.939	-0.908	1.000

Table S8. Explanation of variance.

Component	Initial eigenvalue			Extract square and load		
	Total	Variance (%)	Accumulation (%)	Total	Variance (%)	Accumulation (%)
1	6.763	42.266	42.266	6.763	42.266	42.266
2	2.551	15.943	58.209	2.551	15.943	58.209
3	1.919	11.997	70.206	1.919	11.997	70.206
4	1.587	9.916	80.122	1.587	9.916	80.122
5	0.953	5.957	86.079			
6	0.630	3.936	90.015			
7	0.519	3.245	93.260			
8	0.419	2.616	95.876			
9	0.293	1.833	97.710			
10	0.167	1.046	98.756			
11	0.091	0.570	99.326			
12	0.051	0.321	99.647			
13	0.040	0.250	99.897			
14	0.015	0.092	99.989			
15	0.002	0.011	100.000			
16	0.000	0.000	100.000			

Table S9. Component and score coefficient matrix. (LOD, loss on drying; BFE, basic flowability energy; SI, stability index; FRI, flow rate index; SE, specific energy; CBD, conditioned bulk density).

Variable		Component				Score coefficient			
		1	2	3	4	1	2	3	4
LOD	a_1	0.106	-0.605	0.315	0.599	0.016	-0.237	0.164	0.377
pH	a_2	-0.424	-0.073	0.417	0.153	-0.063	-0.029	0.217	0.096
D10	a_3	0.614	0.613	0.009	0.219	0.091	0.240	0.005	0.138
D50	a_4	0.721	0.573	-0.056	0.208	0.107	0.225	-0.029	0.131
D90	a_5	0.749	0.494	-0.107	0.199	0.111	0.194	-0.056	0.125
Bulk density	a_6	0.842	-0.255	0.400	-0.147	0.125	-0.100	0.208	-0.092
Tapped density	a_7	0.066	0.369	0.836	0.135	0.010	0.145	0.436	0.085
True density	a_8	0.321	-0.312	-0.481	0.376	0.047	-0.122	-0.250	0.237
Hausner ratio	a_9	-0.780	0.469	0.277	0.192	-0.115	0.184	0.144	0.121
Compressibility	a_{10}	-0.750	0.527	0.223	0.222	-0.111	0.207	0.116	0.140
Porosity	a_{11}	-0.834	0.243	-0.422	0.164	-0.123	0.095	-0.220	0.103
BFE	a_{12}	0.656	0.315	-0.272	0.394	0.097	0.123	-0.142	0.248
SI	a_{13}	-0.009	-0.489	0.132	0.726	-0.001	-0.192	0.069	0.458
FRI	a_{14}	-0.878	0.026	0.119	-0.076	-0.130	0.010	0.062	-0.048
SE	a_{15}	-0.787	-0.093	-0.075	0.105	-0.116	-0.037	-0.039	0.066
CBD	a_{16}	0.781	-0.171	0.354	-0.283	0.115	-0.067	0.184	-0.179

Table S10. Principal component scores and CQAs. (F1-F4 are four PCs scores).

	F1	F2	F3	F4	Dissolution 5min (%)	Dissolution 10min (%)	Dissolution 15min (%)
A1	39.93	7.14	-58.12	112.64	87.13	88.10	99.70
A2	59.92	11.23	-65.08	135.10	86.77	87.39	98.72
A3	39.62	7.71	-47.39	98.28	82.99	85.72	96.26
B1	41.78	7.29	-61.41	122.94	90.65	90.56	102.24
B2	64.39	11.95	-68.00	145.71	88.54	88.54	100.25
B3	38.29	7.05	-54.74	112.52	86.69	87.21	98.59
B4	-2.96	0.45	-15.76	40.89	83.78	85.89	96.42
B5	54.31	10.68	-54.67	121.51	82.11	84.66	95.65
B6	35.13	6.50	-52.12	108.16	86.16	87.13	98.49
B7	106.35	20.15	-91.48	211.40	89.33	88.62	100.50
B8	115.59	22.16	-92.88	221.32	87.13	87.74	99.36
B9	35.23	6.64	-43.78	95.57	84.04	85.98	96.69
B10	65.74	12.16	-64.46	142.25	88.01	88.36	100.23
C1	86.29	15.79	-83.18	174.05	84.92	86.60	97.74
C2	42.86	7.63	-61.06	121.25	84.57	86.42	97.59
C3	54.75	9.98	-64.21	132.91	87.66	88.27	100.08
C4	49.52	9.49	-55.04	116.67	85.01	86.77	97.90
C5	63.71	12.75	-55.56	128.75	82.81	85.28	96.24
D1	44.68	7.92	-61.78	119.21	84.48	86.16	97.40
D2	40.84	7.11	-64.21	125.60	84.04	85.54	96.50
D3	62.98	11.90	-65.25	133.74	86.25	87.21	98.53
D4	61.80	11.50	-61.81	131.58	86.69	87.30	98.72
D5	54.22	10.58	-56.82	121.96	84.92	86.77	97.87
D6	100.09	18.57	-91.60	196.88	89.42	89.51	101.18
D7	94.15	17.76	-83.13	180.09	84.40	85.98	97.20
D8	44.36	8.00	-60.56	119.54	86.95	88.10	99.54
D9	58.31	10.66	-66.91	138.64	89.68	90.12	101.28
D10	31.50	5.95	-48.98	97.98	85.81	87.04	98.01
D11	-4.91	0.11	-16.59	35.14	78.41	83.87	94.27
D12	60.20	11.86	-57.48	123.97	82.11	85.19	95.84
D13	99.76	19.09	-86.31	199.84	85.98	87.13	98.19
D14	100.62	19.21	-85.32	196.92	86.86	87.48	99.25
D15	39.63	7.63	-45.67	92.37	81.66	84.22	95.47
D16	57.72	10.55	-63.02	134.41	89.77	90.47	101.46
D17	33.34	6.46	-48.82	96.44	84.92	86.25	97.46
D18	59.81	11.15	-66.80	135.94	85.36	86.86	97.94

Table S11. Comparison of the actual and predicted values of dissolution in the PCA-ANN model. (AE, absolute error; RE, relative error).

MCC	Dissolution at 5 min (%)				Dissolution at 10 min (%)				Dissolution at 15 min (%)			
	Actual	Predicted	AE	RE	Actual	Predicted	AE	RE	Actual	Predicted	AE	RE
A1	85.36	84.27	1.09	1.28	86.86	86.10	0.76	0.88	97.94	97.54	0.40	0.41
B3	86.69	85.19	1.50	1.73	87.21	86.94	0.27	0.31	98.59	98.28	0.31	0.31
B6	86.77	85.40	1.37	1.58	87.39	87.18	0.21	0.24	98.72	98.62	0.10	0.10
C3	84.92	84.44	0.48	0.57	86.77	86.15	0.62	0.72	97.87	97.29	0.58	0.60
D8	85.01	84.27	0.74	0.87	86.77	86.09	0.68	0.78	97.90	97.37	0.53	0.54
D12	88.01	88.49	0.48	0.55	88.36	88.23	0.13	0.15	100.23	100.10	0.13	0.13
